# Supplementary material for: How does a recent gender norms scale perform? Exploratory factor analyses among adolescents in Ethiopia and Bangladesh
Source: PLOS Glob Public Health. 2025 Oct 16;5(10):e0005190. doi: 10.1371/journal.pgph.0005190 (PMC12530582; doi:10.1371/journal.pgph.0005190)
Supplement: S1 Table — (DOCX) [file pgph.0005190.s001.docx]

S1 Table: Variable names of the GAGE gender norms items in the Ethiopia and Bangladesh datasets

| **Education domain** | |
| --- | --- |
| **cr_edu_boysch** | **“**If a family can afford for one child to go to secondary school it should be the boy only” |
| **cr_edu_science** | **“**Only boys should learn about science, technology, and math” |
| **cr_edu_girlnohelp** | **“**Girls should be sent to school only if they are not needed to help at home” |
| **cr_edu_raisingvoice** | **“**Girls should avoid raising their voice to be lady like” |
| **cr_edu_boysfeelings** | **“**Boys should be able to show their feelings without fear of being teased” |
| **cr_edu_culture** | **“**Our culture makes it harder for girls to achieve their goals than boys” |
| **cr_edu_eegirlsout** | **“**Adolescent girls in my community are more likely to be out of school than adolescent boys” |
| **cr_edu_girlschoolsent** | **“**Girls in my community are sent to school only if they are not needed to help at home” |
| **cr_edu_girlschoolexpect** | **“**Most people in my community expect girls to be sent to school only if they are not needed at home” |
| **Domain time use** | |
| **cr_tu_statements1** | **“**Girls and boys should share household tasks equally” |
| **cr_tu_statements2** | **“**A woman’s most important role is to take care of her home and cook for her family” |
| **cr_tu_statements3** | **“**A man should have the final word on decisions in his home” |
| **cr_tu_statements4** | **“**Most boys and girls in my community do not share household tasks equally” |
| **cr_tu_statements5** | **“**Most people in my community expect men to have the final word about decisions in the home” |
| **cr_tu_statements6** | **“**Most people in my community do not expect girls and boys to share household tasks equally” |
| **cr_tu_statements7** | **“**Most men in my community are the ones who make the decisions in their home” |
| **Domain financial inclusion and economic empowerment norms** | |
| **cr_fin_girlchance** | **“**Women should have the same chance to work outside of the home as men” |
| **cr_fin_notgood** | **“**Women who participate in politics or leadership positions cannot also be good wives or mothers” |
| **cr_fin_impwsav** | **“**It is important for women and adolescent girls to have their own savings” |
| **cr_fin_eework** | **“**Most women in my community have the same chance to work outside the home as men” |
| **cr_fin_nework** | **“**Most people in my community expect women to have the same chance to work outside the home as men” |
| **Domain marriage and relationships norms** | |
| **cr_mar_girlfriend** | **“**A boy should be able to have a girlfriend if he wants to” |
| **cr_mar_boyfriend** | **“**A girl should be able to have a boyfriend if she wants to” |
| **cr_mar_waitedu** | **“**A girl’s marriage can wait until she has completed secondary school” |
| **cr_mar_eemarryage** | **“**Most adolescent girls in my community marry before the age of 18 years” (legal age) |
| **cr_mar_nemarryage** | **“**Adults in my community expect adolescent girls to get married before the age of 18 years” (legal age) |
| ***cr_mar_adolmarry*** | “Adolescent girls should marry before the age of 18 years (legal age)” |
| ***cr_mar_obeyhus*** | “A woman should obey her husband in all things” |
| **Domain sexual and reproductive health norms** | |
| **cr_srh_proudbod** | **“**Girls should be proud of their bodies as they become women” |
| **cr_srh_controldaught** | **“**Families should control their daughters' behaviors more than their sons” |
| **cr_srh_eecontrolgirls** | **“**Most families in my community control their daughters’ behaviors more than their sons’” |
| **cr_srh_necontrolgirls** | “Most people in my community expect families to control their daughter’s behavior more than their sons” |
| ***cr_srh_sexnoresp*** | “A woman who has sex before she marries does not deserve respect” |
| ***cr_srh_usebirthcon*** | “It is appropriate for an adolescent female over the age of 13 to be using birth control methods such as the inectable or the pill” |
| ***cr_srh_usecontrac*** | “It should be in a woman's control to make a decision about whether or not to use a contraceptive method” |

Items in italics = excluded items from the analysis
