# Supplementary material for: How does a recent gender norms scale perform? Exploratory factor analyses among adolescents in Ethiopia and Bangladesh
Source: PLOS Glob Public Health. 2025 Oct 16;5(10):e0005190. doi: 10.1371/journal.pgph.0005190 (PMC12530582; doi:10.1371/journal.pgph.0005190)
Supplement: Attachment — Submitted filename: PLOS Review Comments - April 2025.pdf [file pgph.0005190.s002.pdf]

**PLOS Review Comments - Factor structure of the GAGE gender norms scale for adolescents in Ethiopia and Bangladesh**

Using the Gender and Adolescence: Global Evidence (GAGE) project datasets collected from 2017-2018 in Ethiopia and Bangladesh, the authors adopt Exploratory Factor Analyses approach to assess the factor structure of the GAGE gender norms scale with a focus on vulnerable adolescents.

The authors provide great insights into quantitative measurement of vulnerable adolescent gender norms using a two-factor level of Individual level gender attitude and community level descriptive and injunctive gender norms; as well as five-factor domains of gender norms on basis of education; time use; financial inclusion and economic empowerment; relationships and marriage; and sexual and reproductive health. I have the following comments that could help strengthen the paper:

1. In the second paragraph of the introduction section, the authors provide identified gap on the lack of quantitative measures to assess agreement with gender norms. However, given gender norms are invisible, un/spoken rules on how to behave, this also requires qualitative assessments around them. The paper would benefit from provision of insights on what has been done on qualitative measurements of gender norms, the gaps with such measurements that warranty the need for quantitative measures of gender norms as per the current study.
2. In the materials and methods section, the authors note that GAGE project data captured diversity data of the vulnerable adolescents. Are there any variations across the five-factor level analysis based on the differential nature of adolescents' vulnerability characterised by disabilities; adolescents who married before age 18; refugees; adolescent mothers and out-of-school youth for Ethiopia and Bangladesh. This could be provided in the results section. In case the data was not analysed across such dimensions, this could be highlighted in the discussion and study limitation sections to help strengthen the paper.

Other minor suggested edits for typos are provided as comments in the attached paper. Looking forward to seeing this amazing research work published in PLOS Global Public Health!
