# Supplementary material for: How does a recent gender norms scale perform? Exploratory factor analyses among adolescents in Ethiopia and Bangladesh
Source: PLOS Glob Public Health. 2025 Oct 16;5(10):e0005190. doi: 10.1371/journal.pgph.0005190 (PMC12530582; doi:10.1371/journal.pgph.0005190)
Supplement: Attachment — Submitted filename: Dr Sandra Reviews for PLOS Global Public Health.docx [file pgph.0005190.s004.docx]

Review of the paper about “Assessing the factor structure of the GAGE gender norms scale for adolescents: Exploratory Factor Analyses in datasets from Ethiopia and Bangladesh

**Short title**: Factor structure of the GAGE gender norms scale for adolescents

**General comments of the paper:**

The paper on assessing the factor structure of the GAGE gender norms scale read well and is well structured. The work is significant as it explored how gender norms impact adolescents in Ethiopia and Bangladesh. The study aim was clear and was about to validate the GAGE scale, which measures gender norms and their influence on adolescents' lives. The findings inform socio-cultural factors that shape gendered experiences during adolescents especially those with particularities. However, throughout the paper, these considerations were left behind though they were the focus.

Authors can improve their paper by providing the light on the items to be removed and why, show the gaps these items may bring when the GAGE is used as it was developed.

Finally, provide the importance of the final GAGE scale and its generalizability.

**Points by points**

**Pre-section: The Title**

The title is too long (The normal length shouldn’t go beyond 15 words and so on) and the GAGE word is not speaking itself in the title (I have seen that it is the abbreviation of Gender and Adolescence: Global Evidence)

You can go by “*Gender and Adolescents Global Evidence: Exploratory Factor Analyses of Gender norms in Ethiopia and Bangladesh*” Please review if it fits.

**Section 1: Abstract**

**The background section** in the abstract is informative. The authors can add also what say literature about the importance to include gender norms in the adolescent’s health trajectories before entering to the Lower- and Middle-income countries to explain how the study relates to the previously published research?

Does the gap remain in the quantitative scales? What qualitative perspective brought in the literature so far? Clarify the difference here or change the language of quantitizing.

The authors also can explain in full all abbreviations and keep the abbreviations in the main text (the body of the paper).

**Method:** The items composed datasets are not clear for readers. Are these datasets relating to the adolescents as the general population? What age do you consider as adolescent for this study? What was the main test for which outcomes? Clarify and define the research question!!!!!

**Results**: As the method was not clearly showing the main test, variables and outcome measures, this may be hard for readers to discover the real meaning of the results within the abstract. This should be concise.

**Conclusion**: This conclusion is clear from the unclear results. The authors may adapt the conclusion with the results presented in the abstract. Also, they can define the suitability in this context as only the word does not clarify what are the five domains in the abstract.

Also, the authors may add the purpose of the study if it was to refine the datasets in order to define the gender norms in both countries

**Section 2: Main document**

1. **Introduction:**

**Lines 68-74:** The paragraph is nice, the authors need to add what literature say about this before entering to what is missing (Gaps identified).

**Lines 75-:** Do speak only on the quantitative measures, what about qualitative? The authors can suggest literature review about this.

**Lines 85-86** highlight the existing gender norms scales, could you show the gaps about how they were conceptualized and the impacts they have on gender norms for readers???

**Lines 142-148**: The authors highlighted the aim as to explore the not yet validated GAGE gender norms statistically which are published already. Clarify the readers that though the previous gender norms are published, but they were not probably tested for their psychometric measurement or show the low internal consistency which motivated you to explore further statistically.

**Lines 147-148**: Share the results from this computation

**No research question defined apart from the purpose**

2. **Materials and Methods**

**Line 150: Ethical consideration**

Talk about the data handling privacy though the study did not require ethical clearance.

**Line 153: Data**

**Lines 154-157:** Explain that you did not correct any data; you used the existing program’s data to shape the GAGE (Declare any relationship with you and the project and possible conflict of interest according to the journal guidelines).

**Lines 157-160**: Bring in a new concept which is vulnerability (disability, early marriage, refugees, adolescent mothers and those dropped-out from school) and was not introduced from the beginning even in the abstract. Be consistent throughout and this consideration should be explained and tied from the beginning.

**Line 171: Measures**

In this part, there are two outcome measures. The authors may opt to define first these two and their compositions to clarify the readers what are they.

**Lines 174-175**: Where comes education domain??? Name all domains and explain all including the items under each domain (you can simply them in a table).

**Lines 187-190**: Clarify the questions in the brackets

**Line 191:** Exploratory analysis

**Lines 192**: Explain the choice of using exploratory analysis

**Line 196-197**: Explain the exact test for suitability and the level of acceptability for the suitability (Provide interval measures)

**Lines 205-207**: What are the results from computation before refining the five-factor solution? Explain for readers

**Lines 209-212**: For the cut-off, how many variables eliminated as fallen under or equal to cut-off measure, how many times did you repeated the process to arrive at the acceptable loading?

**Line 213: Results**

**Lines 215-219**: Explain the difference in the sample for both countries. Why don’t you consider equal sample size for the two countries (equal male and equal female)?

Explain if the table was generated by the authors or it is from the GAGE project

**Lines 222-232**: Some information is repeated from above; we computed Bartlett’s test…we refined the factors… (line 231). The author can reduce the duplicates in the sections.

**Line 239**: Two factor solution: Is this the final table after all loadings? The value of less than 0.30 is prevalent; what is your take? Especially in grey color. Will this remain? Remember the cut-off.

Revise all tables and define the cut-off from within.

I am also afraid of the influence of the high number (N) within the population which may influence the decision.

**Line 323: Refined five-factor solution**

For the refined five-factor solution, explain whether the removed items in Ethiopia were also removed from Bangladesh dataset.

Any common cumulative variance for both countries???

Explain table 8 with similarities and differences for both countries

**Line 387: Discussion**

Good discussion flow. The authors can separate all five domains in their discussion for a deep reading with clarity.

**Line 405**: As evidence were observed in the other countries rather than Ethiopia and Bangladesh, the discussion here can focus on the important findings from this study

**Line 457: Strengths and Limitations**

Line 456: Speak on the limitation about the triangulation and generalizability of the present findings in the GAGE countries rather than Ethiopia and Bangladesh as some items were removed

**Line 485: Conclusion**

Revise the entire conclusion to show the importance of the work done so far.
