# Supplementary material for: How does a recent gender norms scale perform? Exploratory factor analyses among adolescents in Ethiopia and Bangladesh
Source: PLOS Glob Public Health. 2025 Oct 16;5(10):e0005190. doi: 10.1371/journal.pgph.0005190 (PMC12530582; doi:10.1371/journal.pgph.0005190)
Supplement: Attachment — Submitted filename: Response to reviewers_PLOS Global Public Health_v3.docx [file pgph.0005190.s006.docx]

**Response to reviewers**

Dear Editorial Office and Reviewers,

Thank you for the opportunity to revise this manuscript. You will find our point-by-point responses to your revision requests below.

Kind regards,

The team

Reviewer 1:

General comments:

Overall, this is a well written manuscript evaluating the psychometric structure of the GAGE gender norms scale for adolescents in Ethiopia and Bangladesh by applying Exploratory Factor Analysis to test its proposed two- and five-factor models. The study identifies useful findings for a refined five-factor structure, noting that based on a reduced set of items, it better captures gender norms in Ethiopia and Bangladesh than the original two-factor model. The study suggests the need for adapting and further validating the GAGE gender norms scale for these and similar cultural contexts. Some revisions are proposed and detailed below.

For a lay audience it would be useful to clarify in the distinction of gender norms (re: stereotypes, roles) & rationale for EFA.

We have included definitions for the two terms: "The conceptualisation is particularly inconsistent when it comes to distinguishing gender norms from related concepts such as gender roles or gender stereotypes. The former refers to “beliefs about appropriate roles for males and females regarding the division of paid labor, homework, and childcare” (Davis & Greenstein, 2009) and the latter “are widely held, generalised assumptions regarding common traits (including strengths and weaknesses), based on group categorisation” (12, 13)."

**Introduction:**

Line 94: If possible, it would be helpful to expand on the effects of gender norms on morbidity and mortality during adolescence. At present, the statement is vague with no direct substantiation.

We have deleted the statement on depression and have included a stronger example: "Gender norms also have a notable effect on morbidity and mortality during adolescence. For example, body dissatisfaction as part of gender norm concepts can be associated with gender-related motives of self-harm (14–16), such as exposure to bullying, victimisation, and sexual/physical abuse (14). Self-harm, in turn, is associated with an increased risk for suicide (17)."

Line 112: Are all 8 items excluded from this adaptation tied back to the topics of intimate relationships, gender-based violence, and sexual behaviours enumerated above?

The 16 remaining items still represent the four domains of the GEM scale: violence, reproductive health and disease prevention, sexuality, and domestic chores and daily life. We have specified the exact four domains in the manuscript, and have highlighted that the remaining 16 items are still reflected across the 4 domains: "For example, the GEM scale was originally developed for individuals aged 15-24 years and covers the four domains of violence, reproductive health and disease prevention, sexuality, and domestic chores and daily life (23, 4). A study in Uganda showed that an adaptation, which includes only 16 of the original 24 items across the four domains is needed when validating the GEM scale for younger adolescents (20, 4)."

It may be useful to detail more elaborately the concern for gender norm scales developed in the Global North; without due consideration for marginalized youth (lines 119-124), given it defines the research gaps aimed to be filled by the GAGE project.

We have expanded on this concern: "Another concern is that most of those scales have been developed in the Global North. This highlights a research gap for adolescents in Lower and Middle-Income (LMI) countries with different cultural and socioeconomic contexts. Moreover, this not only influences who and what is measured, but also risks perpetuating neocolonial biases and assumptions (9, 27). Additionally, the validity of gender norms scales among marginalized or minoritized adolescents is even less understood (7). This is because socio-structural disadvantages, such as social class, race, ethnicity, migrant or refugee status, (dis)ability, religion, and area of residence, are areas affected by gender norms (28). Thus, evidence on the exact pathways of gender norms to health is scarce and the understanding of how adolescents perceive gender norms in cross-cultural settings is limited (4)."

Line 125: The GAGE project is the largest longitudinal study focused on adolescents in the Global South, but there is no mention of this scope. Moreover, there is a strong emphasis on adolescents and young key populations (ie. out of school; with disabilities; married under 18, with greater risk for early childbearing; conflict-affected) but no clarification of such detail in the introduction.

We have slightly adapted this sentence to: "The Gender and Adolescence: Global Evidence (GAGE) project as the largest longitudinal study on adolescents aims to address these research gaps and in vulnerable adolescents in the Global South (e.g., with disabilities, out of school, refugees, married under age 18 etc.) and in the conceptualisation of gender norms."

Line 127: If possible, it would be informative to expand on what the GAGE project has drawn from prior scales, and which items have been newly introduced.

We have added another sentence to the end of this paragraph in which we refer to the publication by Baird for a more detailed overview: "The GAGE project has developed a gender norms scale which includes the aforementioned social norms theory and draws on items from multiple scales: the GAS, the GEM, the IMAGES, and the GNI scale. Some items in the GAGE gender norms scale were also newly introduced. They stem from previously conducted qualitative interviews in the GAGE project (26). Further details of the individual items and the scales from which they were drawn can be found in Table A.5 in the publication by Baird et al. (26)."

Line 134: If possible, could you clarify what you mean by physical and mental health? The GAGE project is described as holistic in approaching adolescent well-being more broadly, so there requires clarity either on their more limited scope of impact, or elaboration on the facets of physical and mental health referred to here.

In this sentence, we are referring to the publication of Baird et al. (2019) on which our research objective is based. They used the GAGE gender norms scale to predict physical and mental health in Bangladesh and Ethiopia, but the scale has not been validated for these two countries yet. We now have added "in their publication" and their reference to clarify this: "As part of the GAGE project, Baird et al. (2019) formed two separate gender norm scores from the GAGE gender norms scale, one for individual-level gender attitudes and the other for perceived community-level gender norms. In their publication, they used these scores to predict physical and mental health in Bangladesh and Ethiopia (26). "

Line 138: The GAGE project has 6 domains (education and learning; health, nutrition, and sexual and reproductive health; bodily integrity and freedom from violence; psychosocial well-being; voice and agency; and economic empowerment) - can you clarify whether the project went on to change these domains, or whether you chose to consolidate them? Can you explain this choice?

The domains we talk about in our study are the domains that are integrated specifically in the GAGE gender norms scale to analyse gender norms. We have revised the sentence to highlight this more, by mentioning the GAGE gender norms scale and by adding the reference of Baird et al. (2019): "Importantly, the gender norms items in the GAGE gender norms scale are further divided into five domains : (1) education; (2) time use; (3) financial inclusion and economic empowerment; (4) relationships and marriage; and (5) sexual and reproductive health domain (26)."

**Materials and Methods:**

Line 155: Can you clarify whether the study was limited to adolescents and their caregivers? To my understanding, the study was meant to consult adults across the adolescents' communities.

We had particularly pointed out caregivers due to the "caregivers” datasets, but we agree that it is important to mention the other stakeholders in the adolescents' communities. We have revised the sentence to: "GAGE follows 18,000 adolescents aged 10 to 19 years as well as other community members over 10 years from 2015 to 2024 (31)."

Line 157: The study was intended to focus on ayKP, as opposed to only purposefully including them - not to simply capture diversity but to fill the gap in which they are excluded from the body of research made publicly available. Clarification to this statement would be beneficial.

We have revised this sentence to: "The project aims to discover ‘what works’ (35) for adolescents considered the most vulnerable. The quantitative survey includes adolescents with disabilities, adolescents who were married before the age of 18, refugees, adolescent mothers and out-of-school youth in the six LMI countries included in the project to address the paucity of data on vulnerable adolescents (35). Besides Rwanda, Nepal, Jordan and Lebanon, the survey also covers Ethiopia and Bangladesh (32, 35)."

**Discussion:**

Line 428: It may be of value to clarify the point around control, and where it could have been more closely aligned to bodily autonomy.

We have revised this paragraph, which now reads as follows: "In particular, the sexual and reproductive health domain includes one item about how girls perceive their bodies in the transition to womanhood. The remaining items ask about control (e.g., “Families should control their daughters' behaviors more than their sons”), which is not specifically related to sexual and reproductive health. Although sexual and reproductive health questions should be asked with caution when surveying young adolescents, this domain does not cover the content it suggests. To align the questions on control more closely with the domain of sexual and reproductive health, specific behaviours could be asked, such as the choice of contraceptive method or the decision when to have children, etc."

Line 456: While there is mention of cultural contexts nationally, what are the considerations for how items may need to be refined to adapt to regions within a country?

We have added the following sentences: "Depending on the extent of differences in attitudes to gender norms between different groups of adolescents or regions within a country, it may be useful to conduct EFA separately for subgroups. For example, the Afar and Oromia regions in Ethiopia have been identified for their different and deeply entrenched gender norms (37). It is possible that similar subgroups of adolescents in different countries could have more similar attitudes to gender norms than different subgroups within a country. When conducting research on a subpopulation, it is recommended to use the list of items that fits best for that subpopulation (20)."

**Reviewer 2:**

The submitted manuscript presents a relevant and timely contribution to the study of gender norms among adolescents in low- and middle-income (LMI) countries, particularly through the psychometric evaluation of the GAGE gender norms scale. The use of datasets from both Ethiopia and Bangladesh strengthens the cross-cultural relevance of the findings.

Strengths: The methodological approach, including the use of Exploratory Factor Analysis (EFA) with principal axis factoring and oblique rotation, is appropriate and well executed. The paper contributes meaningfully to the literature by critically examining the domain structure of a widely used tool (GAGE gender norms scale) and proposing refinements grounded in empirical data. The authors provide detailed rationale for each step in refining the factor model and appropriately justify decisions to exclude variables.

Concerns and Suggestions:

Conceptual Framing and Clarity: The manuscript would benefit from a clearer conceptual explanation of why the two-factor model (individual vs. community level) was initially considered and why it failed empirically. While the results are clearly presented, additional theoretical reflection on this mismatch would enhance the discussion.

We have revised how we present the objective of our analysis at the end of the introduction: "Our analysis draws on the paper of Baird et al. (32) in which the GAGE gender norms scale was introduced. Our aim in this study is to explore the psychometric measurement of the statistically not yet validated GAGE gender norms scale using Exploratory Factor Analysis (EFA). More specifically, we imply a supposed two- and five-factor structure in our EFA, which is in accordance with (i) Baird et al.’s (32) distinction between individual-level gender attitudes and perceived community-level gender norms (=two factors), and (ii) the categorisation of the gender norms items into five domains (=five factors) present in the GAGE data. "

We have also adapted the first paragraph of the discussion section to clarify why we considered the 2-factor solution and to summarise the reasons why it empirically failed: "To assess whether the items and domains of the GAGE gender norms scale contribute to explaining gender norms and attitudes in Ethiopia and Bangladesh, we explored the implied two- and five-factor structures (both in the GAGE gender norms scale and in Baird et al.’s analysis (32)) using EFA. The two-factor structure, distinguishing individual-level gender attitudes and community-level gender norms, showed an unsatisfactorye model fit in both datasets due to the pattern of factor loadings not reflecting the individual-community distinction as well as low communalities, high uniqueness values and low cumulative variance explained."

In the discussion, we have added another potential reason for why the 2-factor solution empirically failed: "Another reason may be that the multidomain structure of the GAGE gender norms scale cannot be captured by a two-factor solution as this would oversimplify the complex structure of the gender norms items."

More elaboration is needed on the implications of the low communalities and uniqueness values. For example, what does this say about the conceptual soundness or cultural validity of certain items?

EFA Interpretation: While the iterative refinement process is rigorous, it is notable that the final refined five-factor solution for Bangladesh retains only 15 of 30 items, and for Ethiopia, 17 items. This may reduce the scale's comprehensiveness. I recommend more discussion on the trade-off between parsimony and content validity and whether some of the removed items might be culturally rephrased rather than excluded. The cumulative variance explained (especially in the initial solutions) is quite low. The authors note this, but additional reflection on the adequacy of these thresholds in social science research (and any regional/contextual considerations) would improve the justification.

We have added a full paragraph to the limitations section to explain these implications: "To note, factor structures were interpreted based on their factor loadings, communalities, and uniqueness values, as well as their cumulative variance explained. Gender norm scales should not be developed based on statistical findings alone, but should also incorporate theoretical and conceptual considerations. Particularly the 15 and 13 excluded items in the refined five-factor solutions should be culturally and linguistically rephrased to increase cultural validity and avoid misinterpretations. Moreover, items with lower communalities may not align well with the intended domain or categorisation, or may overlap poorly with other items within the same domain. These items may need to be adapted or assigned to another domain and only items without strong theoretical rationale may need to be excluded. The rather low cumulative variances explained, in the initial factor solutions, may be attributed to the cultural complexity, context sensitivity, and the multidimensional nature of gender norms. The cultural heterogeneity within Bangladesh and Ethiopia, as well as literacy and interpretation issues among marginalised adolescents in LMI settings may also have contributed. In social sciences, the cumulative variance explained sciences tends to be rather low, particularly when attitudes or perceptions are studied (40)."

Recommendations for Future Research: The conclusion rightly calls for Confirmatory Factor Analysis (CFA), but the authors could also discuss other approaches, such as Item Response Theory (IRT), or qualitative validation (e.g., cognitive interviewing), especially for age- and culture-appropriate adaptation of items.

Thank you for this contribution. We have added these suggestions to this paragraph: "Besides further refining the GAGE gender norms items, conducting CFA or Item Response Theory could be necessary next steps to validate the scale. Further qualitative validation (e.g., cognitive interviewing) could support the process of refining the GAGE gender norms scale and enhance the age- and culture-appropriate adaptation of items."

Language and Structure: Overall, the manuscript is well written, but a few sentences in the results section are densely packed and could benefit from restructuring for clarity, particularly when presenting loadings and explaining the rationale for item exclusion.

We have included small changes throughout the results section to reduce densely packed sentences and to clarify the rationale for item exclusion, for example (refined five-factor solutions): "To refine the initial five-factor solution for Ethiopia, we removed six variables that failed to load onto any of the five factors (cr_fin_impwsav, cr_edu_raisingvoice, cr_mar_waitedu, cr_edu_eegirlsout, cr_edu_boysfeelings, cr_srh_proudbod). The exclusion of these items slightly reduced the KMO from 0.77 to 0.75. Thereafter, we removed another three variables that did not load onto any factor in the reduced set of items (cr_tu_statements1, cr_mar_eemarryage, cr_mar_nemarryage). This resulted in all variables loading onto at least one of the five factors. Further exclusion of items did not result in any additional change in the KMO. Finally, we excluded four more variables due to low communalities (<0.2) and factor loadings (<0.3) (cr_fin_notgood, cr_fin_girlchance, cr_edu_culture, cr_tu_statements4), resulting in a KMO of 0.71. The final set of 17 variables is shown in Table 6."

Ethical and Publication Standards: I found no concerns regarding research ethics, dual publication, or competing interests. The use of secondary data has been appropriately acknowledged and justified.

Recommendation: I recommend a minor revision. With improved theoretical framing and discussion of methodological implications, this paper will make a valuable contribution to global adolescent health and gender research.

**Reviewer 3:**

The authors provide great insights into quantitative measurement of vulnerable adolescent gender norms using a two-factor level of Individual level gender attitude and community level descriptive and injunctive gender norms; as well as five-factor domains of gender norms on basis of education; time use; financial inclusion and economic empowerment; relationships and marriage; and sexual and reproductive health.

In the second paragraph of the introduction section, the authors provide identified gap on the lack of quantitative measures to assess agreement with gender norms. However, given gender norms are invisible, un/spoken rules on how to behave, this also requires qualitative assessments around them. The paper would benefit from provision of insights on what has been done on qualitative measurements of gender norms, the gaps with such measurements that warranty the need for quantitative measures of gender norms as per the current study.

We agree. We have included the following paragraph along with further small changes thereafter to ameliorate readability. " Gender norms have gained recognition as a public health priority, which increased scientific efforts to adequately measure gender norms (3). While qualitative methods are typically used to i) gain deeper insights about attitudes toward gender norms, and ii) to inform scale development (4), quantitative gender norms scales serve to measure gender norms as an outcome and as an important factor to behaviour change and health outcomes (3). They are also used to identify and advance interventions that effectively tackle inequitable gender norms (5). However, in the translation of qualitative findings to quantitative scales, theoretical constructs (e.g., social norms theory) have so far not been adequately incorporated (4, 6)."

In the materials and methods section, the authors note that GAGE project data captured diversity data of the vulnerable adolescents. Are there any variations across the five-factor level analysis based on the differential nature of adolescents’ vulnerability characterised by disabilities; adolescents who married before age 18; refugees; adolescent mothers and out-of-school youth for Ethiopia and Bangladesh. This could be provided in the results section. In case the data was not analysed across such dimensions, this could be highlighted in the discussion and study limitation sections to help strengthen the paper.

We have not done such analysis. Also based on another reviewers' suggestions, we have included the following sentences on the generalisability of the study findings: " Depending on the extent of differences in attitudes to gender norms between different groups of adolescents or regions within a country, it may be useful to conduct EFA separately for subgroups. For example, the Afar and Oromia regions in Ethiopia have been identified for their deeply entrenched gender norms (31). It is possible that similar subgroups of adolescents in different countries could have more similar attitudes to gender norms than different subgroups within a country. While it is recommended to use the list of items that fits best for the specific country or subpopulation (e.g., 15 items for the Bangladesh and 17 items for the Ethiopia dataset), it might be beneficial to use the same set of items when conducting comparisons between countries or subgroups (e.g., 17 items for both datasets) (20)."

We also included the following sentence in the limitations: "Moreover, we did not analyse the factor structures between different subgroups of vulnerable adolescents."

Other minor suggested edits for typos are provided as comments in the attached paper. Looking forward to seeing this amazing research work published in PLOS Global Public Health!

We have integrated the suggestions in the revised manuscript. Concerning your suggestion on SDG 5, which is named “inequality” instead of “inequity”: we prefer to use the term “inequity” consistently throughout the manuscript. To avoid introducing yet another gender-related term in our manuscript (and thereby having to distinguish between “inequity” and “inequality”), we have avoided to use the term “inequality” when referring to SDG 5. This is why we specifically phrase the sentence so that we do not state that the SDG 5 is named “inequity” but rather that it drives efforts to reduce inequities. This can be supported by several indicators of SDG 5 that also tackle gender inequities (e.g. discrimination, violence, equal access etc.)."

**Reviewer 4:**

General comments of the paper:

The paper on assessing the factor structure of the GAGE gender norms scale read well and is well structured. The work is significant as it explored how gender norms impact adolescents in Ethiopia and Bangladesh. The study aim was clear and was about to validate the GAGE scale, which measures gender norms and their influence on adolescents' lives. The findings inform socio-cultural factors that shape gendered experiences during adolescents especially those with particularities.

However, throughout the paper, these considerations were left behind though they were the focus. Authors can improve their paper by providing the light on the items to be removed and why, show the gaps these items may bring when the GAGE is used as it was developed. Finally, provide the importance of the final GAGE scale and its generalizability.

The Title: The title is too long (The normal length shouldn’t go beyond 15 words and so on) and the GAGE word is not speaking itself in the title (I have seen that it is the abbreviation of Gender and Adolescence: Global Evidence). You can go by “Gender and Adolescents Global Evidence: Exploratory Factor Analyses of Gender norms in Ethiopia and Bangladesh” Please review if it fits.

We have revised the title accordingly: "How does a recent gender norms scale perform? Exploratory Factor Analyses among adolescents in Ethiopia and Bangladesh”

Section 1: Abstract

The background section in the abstract is informative. The authors can add also what say literature about the importance to include gender norms in the adolescent’s health trajectories before entering to the Lower- and Middle-income countries to explain how the study relates to the previously published research?

We have added to the first sentence in the introduction to highlight the importance of gender norms in the adolescent's health trajectory: " Inequitable gender norms shape adolescents’ perceptions and behaviours, increasing the risk for adverse health outcomes as adults. However, there is a lack of gender norms scales able to measure and effectively reduce inequitable gender norms, particularly in Lower and Middle-Income countries."

Does the gap remain in the quantitative scales? What qualitative perspective brought in the literature so far? Clarify the difference here or change the language of quantitizing.

We have deleted the word "quantitative" to put more emphasis on gender norms scales.

The authors also can explain in full all abbreviations and keep the abbreviations in the main text (the body of the paper).

We have made sure that we introduced all used abbreviations in the abstract.The abbreviations used in the abstract are quite long (e.g. Exploratory Factor Analyses; Gender and Adolescence: Global Evidence) and would make the abstract quite lengthy.

Method: The items composed datasets are not clear for readers. Are these datasets relating to the adolescents as the general population? What age do you consider as adolescent for this study? What was the main test for which outcomes? Clarify and define the research question!!!!!

We agree that this information was missing. We have specified the population contained in the datasets, which consists of adolescents aged 10-19 years. We have also clarified that our main statistical method is Exploratory Factor Analysis.

Rather than an explicit research question, we have clarified the aim of the study in the form of an objective. " This scale (i) distinguishes between individual-level gender attitudes and community-level gender norms (2 factors), and (ii) categorises the gender norms items into five domains (e.g. education-related gender norms; 5 factors). As a first step to validating the GAGE gender norms scale, our objective in this paper is to analyse the supposed two- and five-factor structure using GAGE datasets from Ethiopia and Bangladesh."

We have slightly changed the methods section in the abstract: "We performed Explorative Factor Analyses (EFA) using Principal Axis Factoring and oblique rotation. Prior to this, we tested sampling adequacy using Bartlett’s test of sphericity and the Kaiser-Meyer Olkin measure on the GAGE Ethiopia and Bangladesh datasets, which include adolescents aged 10-19. In the EFA, we tested the two-factor structure and refined the initial five-factor structure by removing variables that failed to load onto a factor or exhibited cross-loadings. Next, we removed variables with low communalities (<0.2) and low factor loadings (<0.3).

Results: As the method was not clearly showing the main test, variables and outcome measures, this may be hard for readers to discover the real meaning of the results within the abstract. This should be concise. Also, the authors may add the purpose of the study if it was to refine the datasets in order to define the gender norms in both countries.

As stated above, we have revised the research question and the methods section to improve the understanding of the results and the conclusion.

Conclusion: This conclusion is clear from the unclear results. The authors may adapt the conclusion with the results presented in the abstract. Also, they can define the suitability in this context as only the word does not clarify what are the five domains in the abstract.

We had given an example for one of the five domains in the objective: “…categorises the gender norms items into five domains (e.g. education-related gender norms; 5 factors).”

Section 2: Main document

1. Introduction:

Lines 68-74: The paragraph is nice, the authors need to add what literature say about this before entering to what is missing (Gaps identified).

Thank you. We have included the following paragraph to better connect qualitative and quantitative aspects. This has lead us to revise the subsequent paragraphs as well.

"Gender norms have gained recognition as a public health priority, which increased scientific efforts to adequately measure gender norms (3). While qualitative methods are typically used to i) gain deeper insights about attitudes toward gender norms or ii) to inform scale development (4), quantitative gender norms scales serve to measure gender norms as an outcome and as an important factor to behaviour change and health outcomes (3). They are also used to identify and advance interventions that effectively tackle inequitable gender norms (5). However, in the translation of qualitative findings to quantitative scales, theoretical constructs (e.g., social norms theory) were often not adequately incorporated (4, 6)."

Lines 85-86 highlight the existing gender norms scales, could you show the gaps about how they were conceptualized and the impacts they have on gender norms for readers???

We have included the gaps that can typically be found in existing gender norms scales. This may also apply to the mentioned scales. We have decided not to specifically highlight the gaps in each scale as the main objective of this manuscript is the non-validation of the GAGE gender norms scale.

Lines 142-148: The authors highlighted the aim as to explore the not yet validated GAGE gender norms statistically which are published already. Clarify the readers that though the previous gender norms are published, but they were not probably tested for their psychometric measurement or show the low internal consistency which motivated you to explore further statistically.

We have adapted the following sentences accordingly: "Our analysis draws on the paper of Baird et al. (32) in which the GAGE gender norms scale was introduced. This scale has not yet been adequately statistically validated and makes implicit claims about the factor structure of gender norms that have not been empirically verified. Our aim in this study is therefore to explore the factor structure of the GAGE gender norms scale using Exploratory Factor Analysis (EFA). More specifically, we investigate possible factor structures with two or five factors, which correspond to (i) the distinction between individual-level gender attitudes and perceived community-level gender norms, and (ii) the categorisation of the gender norms items into the five domains present in the GAGE data, respectively."

Lines 147-148: Share the results from this computation. No research question defined apart from the purpose

Regrettably, we are not sure to which computation you are referring to in the first sentence. If you are referring to the computed two- and five-factor structures, these are reported in the results. We do not think it would be appropriate to also report them in the introduction. If you are referring to the low internal consistency of the gender norms scale in the publication of Baird et al., we have decided to only mention the non-validation of the gender norms scale due to our objective of exploring the multi-domain structure of the scale.

Concerning the second sentence, we have clarified the aim of the study in the form of an objective rather than an explicit research question (see earlier comments).

2. Materials and Methods

Line 150: Ethical consideration

Talk about the data handling privacy though the study did not require ethical clearance.

We have slightly adapted the sentence: "Our study did not require ethical approval, as it is based on the analysis of publicly available secondary panel data that does not contain personal identifiers."

Line 153: Data

Lines 154-157: Explain that you did not correct any data; you used the existing program’s data to shape the GAGE (Declare any relationship with you and the project and possible conflict of interest according to the journal guidelines).

As we have stated in the documents of the submission, no authors have competing interests for this study or any involvement or relationship with the GAGE project. We have also not added or changed any measures in the original data.

Lines 157-160: Bring in a new concept which is vulnerability (disability, early marriage, refugees, adolescent mothers and those dropped-out from school) and was not introduced from the beginning even in the abstract. Be consistent throughout and this consideration should be explained and tied from the beginning.

We have now introduced the concept of vulnerability both in the abstract and in the introduction, respectively: "The Gender and Adolescence: Global Evidence (GAGE) project proposes a gender norms scale for adolescents considered vulnerable ".

"The Gender and Adolescence: Global Evidence (GAGE) project as the largest longitudinal study on adolescents aims to address these research gaps in adolescents considered vulnerable in the Global South (e.g., adolescents with disabilities, out of school youth, refugees, married under age 18 etc.) and in the conceptualisation of gender norms."

Line 171: Measures

In this part, there are two outcome measures. The authors may opt to define first these two and their compositions to clarify the readers what are they.

Lines 174-175: Where comes education domain??? Name all domains and explain all including the items under each domain (you can simply them in a table).

Line 132-145 in the introduction section of the original manuscript submission introduce the distinction into individual level and community level gender norms (=2 factors) and all five domains (=5 factors) in the GAGE gender norms scale. We have now repeated this information in the first sentence of the measures section: "The GAGE gender norms scale in the Ethiopia and Bangladesh datasets used by Baird et al. (24) consists of individual-level gender attitudes and community-level gender norms items, both of which are embedded in five content-related domains (education; time use; financial inclusion and economic empowerment; relationships and marriage; sexual and reproductive health)."

Lines 187-190: Clarify the questions in the brackets

We agree that the meaning of the variable names of the GAGE gender norms items is unclear. We have added a table that provides an overview of the variable names of the gender norms items and their meaning in the annexe.

Line 191: Exploratory analysis

Lines 192: Explain the choice of using exploratory analysis

We have slightly adapted the first sentence: "We chose EFA to investigate the underlying factor structures of the GAGE gender norms scale for Ethiopia and Bangladesh."

Line 196-197: Explain the exact test for suitability and the level of acceptability for the suitability (Provide interval measures)

We have provided a short description of the respective test and their level of acceptability: "We used Bartlett’s test of sphericity (test for homoscedasticity based on the Chi-squared distribution) and the Kaiser-Meyer-Olkin (KMO) test (measure of sampling adequacy) to assess the suitability of the datasets for EFA. Datasets are suitable, when the null hypothesis for the first test is rejected and the values for the second test are above 0.5 (29)."

Lines 205-207: What are the results from computation before refining the five-factor solution? Explain for readers

The results for the initial five-factor solution, as well as the results for the refined factor solutions are shown and explained in the results section.

Lines 209-212: For the cut-off, how many variables eliminated as fallen under or equal to cut-off measure, how many times did you repeated the process to arrive at the acceptable loading?

This information is provided in the results section and explained step by step for each result (l. 324-332 for the refined five-factor solution in the Ethiopia dataset and l.349-357 for the Bangladesh dataset (in the original manuscript submission)). In the methods section, we have focused on explaining the procedure.

Line 213: Results

Lines 215-219: Explain the difference in the sample for both countries. Why don’t you consider equal sample size for the two countries (equal male and equal female)? I am also afraid of the influence of the high number (N) within the population which may influence the decision.

Thank you for the question. However, it is somewhat puzzling since we did not collect any of the data ourselves. Instead, as described in the methods, we used a data set collected by other researchers as part of the GAGE project, with which we have no affiliation. Therefore, we had no influence over the sample size. If you are suggesting that we should remove participants from the available dataset at random until we have an equal number of males and females in each country and an equal number of participants between countries, we do not understand why this would be preferable, since the EFAs for each country are independently conducted. Furthermore, it is unclear to us which “decision” you fear may be influenced by the high sample size.

Explain if the table was generated by the authors or it is from the GAGE project

The table was generated by the authors of this manuscript.

Lines 222-232: Some information is repeated from above; we computed Bartlett’s test…we refined the factors… (line 231). The author can reduce the duplicates in the sections.

We have deleted the first sentence. The paragraph now reads as follows: "Before conducting the EFA, we computed Bartlett’s test of sphericity and the Kaiser-Meyer Olkin measure of sampling adequacy (KMO) for both the Ethiopian and Bangladeshi datasets."

We have also shortened the last sentence of this paragraph, which explains the procedure for the refined five-factor structure. It now reads: "In the following, we describe the initial two- and five-factor solutions as well as the factor models of the refined five-factor solutions for Ethiopia and Bangladesh and compare the results between datasets."

Line 239: Two factor solution: Is this the final table after all loadings? The value of less than 0.30 is prevalent; what is your take? Especially in grey color. Will this remain? Remember the cut-off.

Revise all tables and define the cut-off from within.

Indeed, this is the final table for the two-factor solution with all loadings for all gender norms items. For the two-factor solutions, however, we did not use cut-offs, as described in the methods.

Line 323: Refined five-factor solution. For the refined five-factor solution, explain whether the removed items in Ethiopia were also removed from Bangladesh dataset. Any common cumulative variance for both countries??? Explain table 8 with similarities and differences for both countries

For each dataset, we have provided the names of the gender norms items that we removed in the refined factor solution (Lines 324-332 in the original manuscript submission for Ethiopia, and lines 349-357 for Bangladesh). In Table 8, we compare and explain these results. We do not report a common measure for cumulative variance (only as separate measures) as we conducted, analysed, and reported the results of each EFA separately. The table is explained in the text immediately following the table.

Line 387: Discussion. Good discussion flow. The authors can separate all five domains in their discussion for a deep reading with clarity. Line 405: As evidence were observed in the other countries rather than Ethiopia and Bangladesh, the discussion here can focus on the important findings from this study

Line 457: Strengths and Limitations

Line 456: Speak on the limitation about the triangulation and generalizability of the present findings in the GAGE countries rather than Ethiopia and Bangladesh as some items were removed

The removed items indicate that they may not adequately capture the gender norms of adolescents in both countries. This finding could possibly be generalised to other countries with similar cultural contexts. As a consequence, the GAGE gender norms scale should be adapted for Bangladesh and Ethiopia. We cannot make assumptions about other GAGE countries, such as Lebanon, because we have not tested the GAGE gender norms scales in these countries.

However, we have added some reflections around the generalisability of the findings at the end of the discussion section: "Our results may be generalisable to countries with a similar cultural context to Ethiopia and Bangladesh. Depending on the extent of differences in attitudes to gender norms between different groups of adolescents or regions within a country, it may be useful to conduct EFA separately for subgroups. For example, the Afar and Oromia regions in Ethiopia have been identified for their deeply entrenched gender norms (31). It is possible that similar subgroups of adolescents in different countries could have more similar attitudes to gender norms than different subgroups within a country. While it is recommended to use the list of items that fits best for the specific country or subpopulation (e.g., 15 items for the Bangladesh and 17 items for the Ethiopia dataset), it might be beneficial to use the same set of items when conducting comparisons between countries or subgroups (e.g., 17 items for both datasets) (20)."

Line 485: Conclusion

Revise the entire conclusion to show the importance of the work done so far.

We have revised the conclusion to put more emphasis on the important results of our analysis: "Gender norms research faces a number of challenges in defining, measuring, and conceptualising gender norms. This applies particularly to gender norms scales for adolescents. Our results indicate that separating the GAGE gender norms items into a two-factor structure with an individual-community distinction may not reflect the data structure for the Ethiopia and Bangladesh datasets. Instead, a multi-domain structure seems more promising. In fact, only our refined five-factor solutions yielded promising results with a reduced set of items. Due to issues with wording or domain assignment of some gender norms items, we propose an adaptation of the GAGE gender norms scale. This could include a cultural and linguistical adaptation, a stronger theoretical foundation and an improved assignment of items to the different domains. With our EFAs, we contribute to improving the validity of a gender norms scale for adolescents in two LMI countries. A refined gender norms scale could be valuable for developing more effective interventions in Ethiopia and Bangladesh to reduce inequitable gender norms."
