# Supplementary material for: How does a recent gender norms scale perform? Exploratory factor analyses among adolescents in Ethiopia and Bangladesh
Source: PLOS Glob Public Health. 2025 Oct 16;5(10):e0005190. doi: 10.1371/journal.pgph.0005190 (PMC12530582; doi:10.1371/journal.pgph.0005190)
Supplement: Attachment — Submitted filename: PGPH-D-25-00488-Reviewer comments.pdf [file pgph.0005190.s003.pdf]

PLOS Global Public Health

Assessing the factor structure of the GAGE gender norms scale for adolescents: Exploratory Factor Analyses in datasets from Ethiopia and Bangladesh

--Manuscript Draft--

**Manuscript Number:**	PGPH-D-25-00488
**Article Type:**	Research Article
**Full Title:**	Assessing the factor structure of the GAGE gender norms scale for adolescents: Exploratory Factor Analyses in datasets from Ethiopia and Bangladesh
**Short Title:**	Factor structure of the GAGE gender norms scale for adolescents
**Corresponding Author:**	Anita Alaze, M. Sc.<br>Bielefeld School of Public Health: Universitat Bielefeld Fakultat fur Gesundheitswissenschaften<br>Bielefeld, GERMANY
**Order of Authors:**	Anita Alaze
	John Grosser
	Oliver Razum
	Céline Miani
**Keywords:**	Exploratory Factor Analysis; Gender Norms Scale; Categorisation; GAGE; Adolescents; Ethiopia; Bangladesh
**Abstract:**	Background:<br>Gender norms shape adolescents' perceptions and behaviours, influencing health trajectories. Quantitative gender norms scales are lacking to measure and effectively reduce unequal gender norms, particularly in Lower and Middle-Income countries. The Gender and Adolescence: Global Evidence (GAGE) project proposes a gender norms scale for adolescents. This scale (i) distinguishes between individual-level gender attitudes and community-level gender norms, and (ii) categorises the gender norms items into five domains (e.g. education-related gender norms). We here analyse a supposed two- and five-factor structure in the GAGE gender norms scale using GAGE datasets from Ethiopia and Bangladesh.<br>Method:<br>We analyse Explorative Factor Analyses (EFA) using Principal Axis Factoring and oblique rotation. After testing Bartlett's test of sphericity and the Kaiser-Meyer Olkin measure for sampling adequacy in the GAGE Ethiopia and Bangladesh datasets, we further refine the initial five-factor structure by removing variables that fail to load onto a factor or exhibited cross-loadings. Next, we remove variables with low communalities (<0.2) and low factor loadings (<0.3).<br>Results:<br>The EFAs comprised 6,183 observations from the Ethiopian and 2,245 observations from the Bangladeshi dataset. The initial five-factor solution seemed more appropriate than the two-factor solution in both datasets, and only the refined five-factor structure provided a solution in which the items corresponded to the five domains. However, this only applies to 17 and 15 of the original 30 items in Ethiopia and Bangladesh, respectively, and two of the factors only include two variables each.<br>Conclusion:<br>The five-domain structure proved more suitable for the Ethiopian and Bangladeshi contexts than the individual-community distinction. However, this only applies to the refined five-factor solution with a reduced set of items. We thus propose an adaptation for the GAGE gender norms scale in Ethiopia and Bangladesh, and in countries with similar cultural contexts. Conceptual challenges, such as questionable domain assignments, highlight the need to further refine the GAGE gender norms scale and to confirm the results by Confirmatory Factor Analysis.
**Additional Information:**	
**Question**	**Response**

Powered by Editorial Manager® and ProduXion Manager® from Aries Systems Corporation

**Financial Disclosure**<br><br>Enter a financial disclosure statement that describes the sources of funding for the work included in this submission and the role the funder(s) played. This includes grants and any commercial funding of the work or authors.<br><br>This statement will be typeset if the manuscript is accepted for publication.<br><br>*Please review the submission guidelines and the instructions link below for detailed requirements and guidance.*	This paper is part of a PhD and was thereby funded within the scope of the Junior Research Group GendEpi (Gender Epidemiology) within the Department of Epidemiology & International Public Health, School of Public Health, Bielefeld University.
**Competing Interests**<br><br>On behalf of all authors, disclose any competing interests that could be perceived to bias this work.<br><br>This statement will be typeset if the manuscript is accepted for publication.<br><br>*Please review the instructions link below and PLOS Global Public Health's competing interests policy to determine what information must be disclosed at submission.*	The authors declare that they have no competing interests.
**Data Availability**<br><br>Before publication, Authors are required to make fully available and without restriction all data underlying their findings. Please see our PLOS Data Policy page for detailed information on this policy.<br><br>A **Data Availability Statement**, detailing where the data can be accessed, is required at first submission. Insert your	The data on which the results of our study are based are third-party data that are publicly archived by the data provider UK data service. They are available free of charge following registration with the data provider.

Powered by Editorial Manager® and ProduXion Manager® from Aries Systems Corporation

Data Availability Statement in the box below.<br><br>Please see the data reporting section of our submission guidelines for instructions on what you need to include in your Data Availability Statement.<br><br>This statement will be typeset if the manuscript is accepted for publication.<br><br>*PLOS allows rare exemptions to address legal and ethical concerns. If you have legal or ethical restrictions, please use the box below to detail these in full sentences for the Journal team to consider.*	

Powered by Editorial Manager® and ProduXion Manager® from Aries Systems Corporation

Manuscript

Click here to access/download;Manuscript;Manuscript_GAGE_FA_PLOS

Assessing the factor structure of the GAGE gender norms scale for adolescents: Exploratory Factor Analyses in datasets from Ethiopia and Bangladesh

Factor structure of the GAGE gender norms scale for adolescents

Authors: Anita Alaze[1*], John Grosser[2], Oliver Razum[1], Céline Miani[1]

Affiliations:

[1]Bielefeld University, School of Public Health, Department of Epidemiology and International Public Health

[2]Bielefeld University, School of Public Health, Department of Health Economics and Health Care Management

*Corresponding author: anita.alaze@uni-bielefeld.de

Abstract

Background:

Gender norms shape adolescents' perceptions and behaviours, influencing health trajectories. Quantitative gender norms scales are lacking to measure and effectively reduce unequal gender norms, particularly in Lower and Middle-Income countries. The Gender and Adolescence: Global Evidence (GAGE) project proposes a gender norms scale for adolescents. This scale (i) distinguishes between individual-level gender attitudes and community-level gender norms, and (ii) categorises the gender norms items into five domains (e.g. education-related gender norms). We here analyse a supposed two- and five-factor structure in the GAGE gender norms scale using GAGE datasets from Ethiopia and Bangladesh.

Method:

We analyse Explorative Factor Analyses (EFA) using Principal Axis Factoring and oblique rotation. After testing Bartlett's test of sphericity and the Kaiser-Meyer Olkin measure for sampling adequacy in the GAGE Ethiopia and Bangladesh datasets, we further refine the initial five-factor structure by removing variables that fail to load onto a factor or exhibited cross-loadings. Next, we remove variables with low communalities (<0.2) and low factor loadings (<0.3).

Results:

The EFAs comprised 6,183 observations from the Ethiopian and 2,245 observations from the Bangladeshi dataset. The initial five-factor solution seemed more appropriate than the two-factor solution in both datasets, and only the refined five-factor structure provided a solution in which the items corresponded to the five domains. However, this only applies to 17 and 15 of the original 30 items in Ethiopia and Bangladesh, respectively, and two of the factors only include two variables each.

Conclusion:

The five-domain structure proved more suitable for the Ethiopian and Bangladeshi contexts than the individual-community distinction. However, this only applies to the refined five-factor solution with a reduced set of items. We thus propose an adaptation for the GAGE gender norms scale in Ethiopia and Bangladesh, and in countries with similar cultural contexts. Conceptual challenges, such as questionable domain assignments, highlight the need to further refine the GAGE gender norms scale and to confirm the results by Confirmatory Factor Analysis.

Keywords: Exploratory Factor Analysis, Gender Norms Scale, Categorisation, GAGE, Adolescents, Ethiopia, Bangladesh

1 Introduction

Gender inequity hinders the realisation of human rights and health for all. While Sustainable Development Goal (SDG) 5 drives global efforts to reduce gender inequities, persisting gender inequity has been identified as one of the main obstacles hindering further progress on the other SDGs (1). Among the biggest barriers to gender inequity are societal gender norms (1). Gender norms are defined as the "spoken and unspoken rules of societies about the acceptable behaviours of girls and boys […] how they should act, look, and even think or feel" (2).

However, there is a lack of quantitative measures to assess agreement with gender norms. The further development of such gender norms measures is necessary to identify and advance interventions that effectively tackle inequitable gender norms (3). A key research gap around the measurement of gender norms concerns their conceptualisation and validity (4, 5). Various quantitative surveys, such as the World Value Survey and the International Social Survey Programme (5), contain unintentional gender bias in question phrasing and translation, mostly rely on proxies for gender norms, such as social aggregates for individual behaviours and attitudes, and do not contain missing information on gender identity and individuals' gendered experiences (e.g. socially constructed gender roles, gender practices and relationship dynamics) (6).

Various gender norms scales exist, but they use different conceptualisations of gender norms. This can be seen from the different terminologies used in gender norms scales such as the Gender Attitudes Scale (GAS) bynLundgren et al. (7), the International Men and Gender Equality Survey (IMAGES) by Levtov et al., (8), and the G-NORM scale by Sedlander et al. (9). The conceptualisation is particularly inconsistent when it comes to distinguishing gender norms from related concepts such as gender roles or gender stereotypes.

Adolescence has been recognised as a window of opportunity to change inequitable gender norms. It is a critical and intensified period of life (10) in which adolescents' perspectives towards gender norms are shaped. Gender norms also have a notable effect on morbidity and mortality during adolescence. For example, the prevalence of depression, suicidal behaviours and stress-related disorders increases significantly during adolescence (11–13). Gender norms influence the health trajectories of adolescents throughout a lifetime (14). For example, adolescents who adopt gender inequitable attitudes and roles are at greater risk of experiencing adverse gender-related health outcomes as adults (15). This makes adolescence an influential period in which health-related outcomes are predetermined and individual attitudes towards gender norms are formed.

Although some gender norms scales have been specifically developed for or adapted to adolescents, such as the Gender Equitable Men (GEM) Scale by Vu et al. (16), Attitudes Towards Women Scale (for Adolescents) by Galambos et al. (17), and the Adolescent Femininity Ideology Scale by Tolman and Porche (18), the age group of adolescents has so far been greatly overlooked in research (14).

A conceptual challenge with those scales arises through the fact that particularly younger adolescents find it difficult to answer the provided questions. This is because many of them may have not yet engaged in sexual relations or have difficulties imagining abstract situations (10). For example, the GEM scale was originally developed for individuals aged 15-24 years and covers the topics of intimate relationships, gender-based violence, and sexual behaviours (10, 19). A study in Uganda showed that an adaptation of including only 16 of the original 24 items is needed when validating the GEM scale for younger adolescents (10, 16). Although the Gender Norm Instrument (GNI) of the Global Early Adolescent Study (20), and the Male Role Norms Inventory (MNI) – Adolescent, revised by Levant et al. (21) are gender norms scales developed specifically for early adolescence, further research is needed. In particular, because interventions to tackle inequitable gender norms may be more effective at a younger age (22).

Another concern is that most of those scales have been developed in the Global North. This highlights a research gap for adolescents in Lower and Middle-Income (LMI) countries with different cultural and socioeconomic contexts. Additionally, the validity of gender norms scales among marginalized or minoritized adolescents is even less understood (4). Thus, evidence on the exact pathways of gender norms to health is scarce and the understanding of how adolescents perceive gender norms in cross-cultural settings is limited (10).

The Gender and Adolescence: Global Evidence (GAGE) project aims to address these research gaps in adolescents and in the conceptualisation of gender norms, focusing on vulnerable adolescents in the Global South. The GAGE project has developed a gender norms scale which draws on items from multiple scales: the GAS, the GEM, the IMAGES, and the GNI scale. Some items in the GAGE gender norms scale were also newly introduced. They stem from previously conducted qualitative interviews in the GAGE project (23).

As part of the GAGE project, Baird et al. (2019) formed two separate gender norm scores from the GAGE gender norms scale, one for individual-level gender attitudes and the other for perceived community-level gender norms. They used these scores to predict physical and mental health in Bangladesh and Ethiopia. Gender attitudes measure what the adolescent thinks about the gendered attitude while perceived community-level gender norms measure descriptive norms as to “what the [adolescent] believes others do” and injunctive norms as to “what the [adolescent] believes others think that s/he should do” (23, 24). Importantly, the gender norms items are further divided into five domains in the GAGE data: (1) education; (2) time use; (3) financial inclusion and economic empowerment; (4) relationships and marriage; and (5) sexual and reproductive health domain.

Our aim in this paper is to explore the statistically not yet validated GAGE gender norms scale using Exploratory Factor Analysis (EFA). More specifically, we imply a supposed two-factor- and five-factor structure in our EFA, which is in accordance with (i) Baird et al.’s distinction between individual-level gender attitudes and perceived community-level gender

norms (=two factors), and (ii) the categorisation of the gender norms items into five domains (=five factors) present in the GAGE data. Thus, we compute two- and five-factor structures in our EFA, and compare the results between two countries.

2 Materials and Methods

2.1 Ethical approval

Our study did not require ethical approval, as it is based on the analysis of publicly available secondary panel data.

2.2 Data

Our statistical analysis draws on quantitative data from the GAGE project, a mixed-methods longitudinal research and evaluation programme. Across six countries, GAGE follows 18,000 adolescents aged 10 to 19 years as well as their caregivers over 10 years from 2015 to 2024 (25). The project aims to discover 'what works' (26) for the most vulnerable adolescents. The quantitative survey purposefully included adolescents with disabilities, adolescents who married before age 18, refugees, adolescent mothers and out-of-school youth to capture diversity in the six LMI countries covered by the project (26). Besides Rwanda, Nepal, Jordan and Lebanon, the survey also covers Ethiopia and Bangladesh (23, 26).

Following Baird et al.'s analysis, our study draws on the baseline findings collected by the GAGE project in Ethiopia and Bangladesh in 2017 and 2018. Data collection took place in six locations in Ethiopia and in three locations in Bangladesh. While in Ethiopia and one Bangladesh location, a household census was used to identify potential participants, a school-based census was employed in the other two Bangladesh locations (23). Further details on data collection procedures are described in Baird et al., (23).

The data on which the results of our study are based are third-party data that are publicly archived by the data provider UK data service. They are available free of charge following registration with the data provider.

2.3 Measures

The individual-level gender attitudes and the community-level gender norms items of the GAGE gender norms scale in the Ethiopian and Bangladeshi datasets used by Baird et al. (23) are embedded in five content-related domains. An example for the *education* domain is: “Girls should be sent to school only if they are not needed to help at home”. “Girls and boys should share household tasks equally” is an example for the time use domain and “women who participate in politics or leadership positions cannot also be good wives or mothers” is an example for the *financial inclusion and economic empowerment* domain. The *relationships and marriage* domain is captured, for example, by “adolescent girls should marry before the age of 18 years (legal age)” and the *sexual and reproductive health* domain is captured by questions such as “families should control their daughters' behaviors more than their sons”. Table 1 provides an overview of all included gender norms items.

Three items in the *sexual and reproductive health* domain and one item in the *relationships and marriage* domain could not be included as they were only answered by older adolescents. The gender norms items are three-level variables with ‘1’ indicating agreement, ‘2’ indicating partial agreement and ‘3’ indicating disagreement with the statement. Following Baird et al., we recoded all items with a reverse question (cr_edu_boysfeelings, cr_fin_girlchance, cr_fin_impwsav, cr_fin_nework, cr_fin_eework, cr_tu_statements1, cr_mar_girlfriend, cr_mar_boyfriend, cr_mar_waitedu, cr_srh_proudbod) to ensure that agreement suggests a gender-unequal response.

2.4 Exploratory Factor Analysis

We chose EFA for an exploratory analysis of the underlying factor structures of the GAGE gender norms scale for Ethiopia and Bangladesh. We used Principal Axis Factoring and

oblimin rotation (an oblique factor rotation method). Oblique rotation allows non-zero correlation between factors (27), which fits our likely highly correlated gender norms items. We used Bartlett's test of sphericity and the Kaiser-Meyer-Olkin (KMO) test to assess the suitability of the datasets for EFA. We conducted all statistical analyses using the software R (version 4.4.1, R Core Team, 2024).

In their analysis, Baird et al. form two separate gender norm scores (one for individual-level gender attitudes, the other for perceived community-level gender norms) and use these scores as predictors in regression models for physical and mental health (23). Although they do not say so explicitly, this approach seems to imply a supposed two-factor (individual- vs. community-level) structure in the gender norm data.

To explore the validity of this supposed two-factor structure, we first calculated a two-factor solution for both datasets. Thereafter, we computed a five-factor solution (in accordance with the five domains present in the GAGE data). After computing the initial two- and five-factor solutions, we further refined the five-factor solution for both datasets. In particular, we excluded variables that failed to load onto a factor or exhibited cross-loadings. We iteratively refined the model until all remaining variables loaded onto a factor. We then applied cutoffs, removing variables with communalities below 0.2 (28, 29) and factor loadings below 0.3 (30, 29). We repeated this process until all variables loaded onto a factor and did not produce cross-loadings.

3 Results

3.1 Sample characteristics

The Ethiopian sample contained 6,985 and the Bangladeshi sample 2,576 adolescents. After restricting the sample to those adolescents who answered all gender norm questions, the datasets comprise 6,183 individuals for Ethiopia (2,767 boys; 3,416 girls) and 2,245

individuals for Bangladesh (1,104 boys; 1,141 girls). The descriptive statistics of the gender norms items in the two datasets are presented in Table 1.

Table 1: Descriptive statistics of the GAGE gender norms items in the Ethiopian and Bangladeshi datasets.

Gender norms	Sample distribution	
	Ethiopia n (%)	Bangladesh n (%)
Education domain		
**cr_edu_boysch: "**If a family can afford for one child to go to secondary school it should be the boy only"		
Agree	1339 (21.7)	383 (17.1)
Partially agree	669 (10.8)	269 (12.0)
Do not agree	4175 (67.5)	1593 (71.0)
**cr_edu_science: "**Only boys should learn about science, technology, and math"		
Agree	767 (12.4)	192 (8.6)
Partially agree	493 (8.0)	200 (8.9)
Do not agree	4923 (79.6)	1853 (82.5)
**cr_edu_girlnohelp: "**Girls should be sent to school only if they are not needed to help at home"		
Agree	942 (15.2)	267 (11.9)
Partially agree	688 (11.1)	251 (11.2)
Do not agree	4553 (73.6)	1727 (76.9)
**cr_edu_raisingvoice: "**Girls should avoid raising their voice to be lady like"		
Agree	2540 (41.1)	1474 (65.7)
Partially agree	967 (15.6)	222 (9.9)
Do not agree	2676 (43.3)	549 (24.5)
**cr_edu_boysfeelings: "**Boys should be able to show their feelings without fear of being teased"		
Agree	4834 (78.2)	666 (29.7)
Partially agree	692 (11.2)	338 (15.1)
Do not agree	657 (10.6)	1241 (55.3)
**cr_edu_culture: "**Our culture makes it harder for girls to achieve their goals than boys"		
Agree	3061 (49.5)	1302 (58.0)
Partially agree	770 (12.5)	382 (17.0)
Do not agree	2352 (38.0)	561 (25.0)
**cr_edu_eegirlsout: "**Adolescent girls in my community are more likely to be out of school than adolescent boys"		
Agree	3187 (51.5)	488 (21.7)
Partially agree	1105 (17.9)	445 (19.8)
Do not agree	1891 (30.6)	1312 (58.4)
**cr_edu_girlschoolsent: "**Girls in my community are sent to school only if they are not needed to help at home"		
Agree	1302 (21.1)	332 (14.8)
Partially agree	984 (15.9)	330 (14.7)
Do not agree	1897 (63.0)	1583 (70.5)
**cr_edu_girlschoolexpect: "**Most people in my community expect girls to be sent to school only if they are not needed at home"		
Agree	1777 (28.7)	361 (16.1)
Partially agree	1123 (18.2)	354 (15.8)
Do not agree	3283 (53.1)	1530 (68.2)
**Domain time use**		
**cr_tu_statements1:** "Girls and boys should share household tasks equally"		
Agree	4212 (68.1)	1871 (83.3)
Partially agree	778 (12.6)	174 (7.8)
Do not agree	1193 (19.3)	200 (08.9)
**cr_tu_statements2: "**A woman's most important role is to take care of her home and cook for her family"		

Agree	3681 (59.5)	1147 (51.1)
Partially agree	926 (15.0)	325 (14.5)
Do not agree	1576 (25.5)	773 (34.4)
**cr_tu_statements3:** “A man should have the final word on decisions in his home”		
Agree	3537 (57.2)	937 (41.7)
Partially agree	972 (15.7)	326 (14.5)
Do not agree	1674 (27.1)	982 (43.7)
**cr_tu_statements4:** “Most boys and girls in my community do not share household tasks equally”		
Agree	3871 (62.6)	1356 (60.4)
Partially agree	1005 (16.3)	361 (16.1)
Do not agree	1307 (21.1)	528 (23.5)
**cr_tu_statements5:** “Most people in my community expect men to have the final word about decisions in the home”		
Agree	4085 (66.1)	977 (43.5)
Partially agree	1022 (16.5)	454 (20.2)
Do not agree	1076 (17.4)	814 (36.3)
**cr_tu_statements6:** “Most people in my community do not expect girls and boys to share household tasks equally”		
Agree	4169 (67.4)	1264 (56.3)
Partially agree	979 (15.8)	428 (19.1)
Do not agree	1035 (16.7)	553 (24.6)
**cr_tu_statements7:** “Most men in my community are the ones who make the decisions in their home”		
Agree	4085 (66.1)	1091 (48.6)
Partially agree	1041 (16.8)	501 (22.3)
Do not agree	1057 (17.1)	653 (29.1)
**Domain financial inclusion and economic empowerment norms**		
**cr_fin_girlchance:** “Women should have the same chance to work outside of the home as men”		
Agree	5106 (82.6)	1850 (82.4)
Partially agree	577 (9.3)	166 (7.4)
Do not agree	500 (8.1)	229 (10.2)
**cr_fin_notgood:** “Women who participate in politics or leadership positions cannot also be good wives or mothers”		
Agree	1696 (27.4)	653 (29.1)
Partially agree	795 (12.9)	426 (19.0)
Do not agree	3692 (59.7)	1166 (51.9)
**cr_fin_impwsav:** “It is important for women and adolescent girls to have their own savings”		
Agree	5521 (89.3)	1999 (89.0)
Partially agree	470 (7.6)	165 (7.4)
Do not agree	192 (3.1)	81 (3.6)
**cr_fin_eework:** “Most women in my community have the same chance to work outside the home as men”		
Agree	3822 (61.8)	1400 (62.4)
Partially agree	1188 (19.2)	443 (19.7)
Do not agree	1173 (19.0)	402 (17.9)
**cr_fin_nework:** “Most people in my community expect women to have the same chance to work outside the home as men”		
Agree	3606 (58.3)	1432 (63.8)
Partially agree	1324 (21.4)	458 (20.4)
Do not agree	1253 (20.3)	355 (15.8)
**Domain relationships and marriage norms**		
**cr_mar_girlfriend:** “A boy should be able to have a girlfriend if he wants to”		
Agree	3850 (62.3)	1869 (83.5)
Partially agree	669 (10.8)	153 (6.8)
Do not agree	1664 (26.9)	223 (9.9)
**cr_mar_boyfriend:** “A girl should be able to have a boyfriend if she wants to”		
Agree	3487 (56.4)	1821 (81.1)
Partially agree	763 (12.3)	180 (8.0)
Do not agree	1933 (31.3)	244 (10.9)

**cr_mar_waitedu: "**A girl's marriage can wait until she has completed secondary school"		
Agree	4719 (76.3)	1712 (76.3)
Partially agree	698 (11.3)	180 (8.0)
Do not agree	766 (12.4)	353 (15.7)
**cr_mar_eemarryage: "**Most adolescent girls in my community marry before the age of 18 years" (legal age)		
Agree	2534 (41.0)	841 (37.5)
Partially agree	944 (15.3)	442 (19.7)
Do not agree	2705 (43.8)	962 (42.9)
**cr_mar_nemarryage: "**Adults in my community expect adolescent girls to get married before the age of 18 years" (legal age)		
Agree	2325 (37.6)	534 (23.8)
Partially agree	939 (15.2)	438 (19.5)
Do not agree	2919 (47.2)	1273 (56.7)
**Domain sexual and reproductive health norms**		
**cr_srh_proudbod: "**Girls should be proud of their bodies as they become women"		
Agree	4771 (77.2)	1236 (55.1)
Partially agree	523 (8.5)	344 (15.3)
Do not agree	889 (14.4)	665 (29.6)
**cr_srh_controldaught: "**Families should control their daughters' behaviors more than their sons"		
Agree	4123 (66.7)	1392 (62.0)
Partially agree	805 (13.0)	395 (17.6)
Do not agree	1255 (20.3)	458 (20.4)
**cr_srh_eecontrolgirls: "**Most families in my community control their daughters' behaviors more than their sons'"		
Agree	4613 (74.6)	1454 (64.8)
Partially agree	818 (13.2)	421 (18.8)
Do not agree	752 (12.2)	370 (16.5)
**cr_srh_necontrolgirls: "**Most people in my community expect families to control their daughter's behavior more than their sons'"		
Agree	4558 (73.7)	1404 (62.5)
Partially agree	857 (13.9)	462 (20.6)
Do not agree	768 (12.4)	379 (16.9)

3.2 Exploratory Factor Analysis

Before conducting the EFA, we computed Bartlett's test of sphericity and the Kaiser-Meyer Olkin measure of sampling adequacy (KMO) for both the Ethiopian and Bangladeshi datasets. Bartlett's test of sphericity yielded values of $\chi^2(435) = 46514.04$, $p<0.001$ for the Ethiopian dataset and $\chi^2(435) = 13177.59$, $p<0.001$ for the Bangladeshi dataset, indicating that the inter-item correlations were sufficiently large for EFA in both datasets (30).

The overall KMO of the Ethiopian and Bangladeshi datasets was 0.77 and 0.74, respectively, while the KMO of the individual variables ranged from 0.52 to 0.92 and from 0.55 to 0.86, respectively. With values above 0.5, the KMOs suggest adequate samples for conducting an EFA (30). As described in the methods, we then computed the initial two- and five-factor

solutions. We then iteratively refined the factor models of the initial five-factor solutions for Ethiopia and Bangladesh and compared the results between datasets.

3.2.1 Two-Factor Solution

The two-factor EFA for the Ethiopia dataset is shown in Table 2. Of the 30 variables, 14 loaded onto the first factor (threshold 0.30), while only three variables loaded onto the second factor. Of the former, four variables loaded strongly onto the first factor (threshold 0.50), while all three of the latter loaded strongly onto the second factor. Thirteen variables loaded onto neither factor.

Table 2: Two-factor solution for the Ethiopian dataset (N=6,183)

Question content	Factor 1	Factor 2	Communality	Uniqueness
If a family can afford for one child to go to secondary school it should be the boy only	0.52	-0.02	0.27	0.73
Only boys should learn about science, technology, and math	0.51	-0.07	0.25	0.75
Girls should be sent to school only if they are not needed to help at home	0.54	-0.09	0.28	0.72
Girls should avoid raising their voice to be lady like	0.39	0.05	0.16	0.84
Boys should be able to show their feelings without fear of being teased	0.03	-0.11	0.01	1.00
Girls and boys should share household tasks equally	0.23	-0.14	0.06	0.94
A woman’s most important role is to take care of her home and cook for her family	0.43	0.13	0.23	0.77
A man should have the final word on decisions in his home	0.46	0.20	0.29	0.71
Women should have the same chance to work outside of the home as men	0.26	-0.27	0.11	0.89
Women who participate in politics or leadership positions cannot also be good wives or mothers	0.39	0.07	0.16	0.84
It is important for women and adolescent girls to have their own savings	0.23	-0.27	0.10	0.90
A boy should be able to have a girlfriend if he wants to	0.01	-0.21	0.04	0.96
A girl should be able to have a boyfriend if she wants to	0.08	-0.21	0.04	0.96
A girl’s marriage can wait until she has completed secondary school	0.10	-0.17	0.03	0.97
Girls should be proud of their bodies as they become women	0.00	-0.16	0.03	0.97
Families should control their daughters' behaviors more than their sons	0.10	0.54	0.33	0.67
Our culture makes it harder for girls to achieve their goals than boys	0.45	0.02	0.21	0.79
Adolescent girls in my community are more likely to be out of school than adolescent boys	0.30	0.08	0.10	0.90
Girls in my community are sent to school only if they are not needed to help at home	0.58	-0.09	0.32	0.68

Most people in my community expect girls to be sent to school only if they are not needed at home	0.48	-0.04	0.22	0.78
Most boys and girls in my community do not share household tasks equally	0.19	0.20	0.09	0.91
Most people in my community expect men to have the final word about decisions in the home	0.39	0.24	0.25	0.75
Most people in my community do not expect girls and boys to share household tasks equally	0.28	0.22	0.15	0.85
Most men in my community are the ones who make the decisions in their home	0.37	0.26	0.25	0.75
Most women in my community have the same chance to work outside the home as men	0.29	-0.24	0.11	0.89
Most people in my community expect women to have the same chance to work outside the home as men	0.28	-0.22	0.10	0.90
Most adolescent girls in my community marry before the age of 18 years (legal age)	0.39	0.07	0.17	0.83
Adults in my community expect adolescent girls to get married before the age of 18 years (legal age)	0.40	0.06	0.18	0.82
Most families in my community control their daughters' behaviors more than their sons'	-0.01	0.72	0.51	0.49
Most people in my community expect families to control their daughter's behavior more than their sons'	0.00	0.71	0.50	0.50
Cumulative variance explained	11.62%	18.55%		

Communality and uniqueness are rounded to the second decimal place
Green highlighted factor loadings = above 0.50 (strong loading)
Yellow highlighted factor loadings = above 0.30 (weak loading)
Grey highlighted rows = community-level gender norms

Notably, the loading patterns do not correspond to the expected two factors (perceived community-level gender norms and individual-level gender attitudes). In particular, the second factor contains only variables on what was defined as *sexual and reproductive health*, including two community-level variables and one individual-level variable. The first factor also contains both individual- and community-level variables, particularly on *education* and *time use*.

Furthermore, the two-factor solution does not fit the data well. Beyond the high number of variables that do not load onto any factor, the communalities of the variables are low across the board. In fact, only two variables have a communality of more than 0.50. A substantial number of variables has a very high uniqueness (higher than 0.80), indicating that they contribute little to the factor structure. Only two variables have uniqueness values below 0.60. The two factors explained 11.62% and 6.93% of the variance, respectively, for a total of only 18.55%.

In the Bangladeshi dataset, seven variables loaded onto the first factor, three of them strongly. Another seven variables loaded onto the second factor, only a single one of them strongly. The remaining sixteen variables loaded onto neither factor. The first factor contains most of the *education* variables, including some (but not all) individual- and community-level variables. The second factor contains most of the variables (both individual- and community-level) on *sexual and reproductive health*, along with some *time-use* variables and one *education* variable. The two-factor solution for the Bangladeshi dataset is shown in Table 3.

Table 3: Two-factor solution for the Bangladeshi dataset (N=2,245)

Question content	Factor 1	Factor 2	Communality	Uniqueness
If a family can afford for one child to go to secondary school it should be the boy only	0.43	0.01	0.19	0.81
Only boys should learn about science, technology, and math	0.47	0.02	0.23	0.77
Girls should be sent to school only if they are not needed to help at home	0.63	-0.06	0.38	0.62
Girls should avoid raising their voice to be lady like	-0.18	0.37	0.15	0.85
Boys should be able to show their feelings without fear of being teased	-0.23	-0.17	0.10	0.90
Girls and boys should share household tasks equally	0.14	-0.06	0.02	0.98
A woman’s most important role is to take care of her home and cook for her family	0.27	0.35	0.23	0.77
A man should have the final word on decisions in his home	0.37	0.28	0.25	0.75
Women should have the same chance to work outside of the home as men	0.25	0.02	0.07	0.93
Women who participate in politics or leadership positions cannot also be good wives or mothers	0.13	0.17	0.05	0.95
It is important for women and adolescent girls to have their own savings	0.25	-0.06	0.06	0.94
A boy should be able to have a girlfriend if he wants to	0.21	-0.42	0.19	0.81
A girl should be able to have a boyfriend if she wants to	0.20	-0.39	0.16	0.84
A girl’s marriage can wait until she has completed secondary school	0.22	-0.33	0.13	0.87
Girls should be proud of their bodies as they become women	0.01	-0.26	0.06	0.94
Families should control their daughters' behaviors more than their sons	0.04	0.36	0.14	0.86
Our culture makes it harder for girls to achieve their goals than boys	0.12	0.14	0.04	0.96
Adolescent girls in my community are more likely to be out of school than adolescent boys	0.31	-0.13	0.10	0.90
Girls in my community are sent to school only if they are not needed to help at home	0.66	-0.01	0.43	0.57
Most people in my community expect girls to be sent to school only if they are not needed at home	0.57	0.04	0.33	0.67
Most boys and girls in my community do not share household tasks equally	0.00	0.22	0.05	0.95

Most people in my community expect men to have the final word about decisions in the home	0.27	0.37	0.24	0.76
Most people in my community do not expect girls and boys to share household tasks equally	0.01	0.11	0.01	0.99
Most men in my community are the ones who make the decisions in their home	0.18	0.43	0.24	0.76
Most women in my community have the same chance to work outside the home as men	0.15	0.12	0.04	0.96
Most people in my community expect women to have the same chance to work outside the home as men	0.15	0.10	0.04	0.96
Most adolescent girls in my community marry before the age of 18 years (legal age)	0.22	0.19	0.10	0.90
Adults in my community expect adolescent girls to get married before the age of 18 years (legal age)	0.24	0.18	0.11	0.89
Most families in my community control their daughters' behaviors more than their sons'	0.03	0.48	0.23	0.77
Most people in my community expect families to control their daughter's behavior more than their sons'	0.00	0.52	0.27	0.73
Cumulative variance explained	8.53%	15.48%		

Communality and uniqueness are rounded to the second decimal place
Green highlighted factor loadings = above 0.50 (strong loading)
Yellow highlighted factor loadings = above 0.30 (weak loading)
Grey highlighted rows = community-level gender norms

Once again, therefore, the loadings do not correspond well to the individual-community distinction. Furthermore, as in the Ethiopian dataset, the factor solution does not adequately fit the data. Communalities are again low, with no variable having a communality above 0.43. The cumulative proportion of explained variance is only 15.48%, with 8.53% of variance explained by the first and 6.96% by the second factor.

In summary, the two-factor solution is unsatisfactory in both datasets. This indicates that it is not a reflection of the data structure to divide the variables into a community-level and an individual-level factor. Instead, the fact that both datasets yield a factor that contains only or mostly variables from a single domain indicates that a five-factor solution based on these domains may be more appropriate.

3.2.2 Initial Five-Factor Solution

The five-factor solution for the Ethiopia dataset, shown in Table 4, generally supports this notion. Each of the five factors corresponds roughly to one of the five domains. The first factor is strongly loaded onto five out of nine *education* variables, with another *education* variable loading weakly. In contrast, only one non-*education* variable loads (weakly) onto the

first factor. The second factor contains all but one of the *time-use* variables, with most of these variables loading strongly. Two non-*time-use* variables also loaded onto the second factor, though only loaded weakly. Three of four *sexual and reproductive health* variables loaded strongly onto the third factor. The fourth factor includes mostly *financial and economic empowerment* variables, while the fifth factor includes only two variables, both of which belong to the *marriage and relationship* domain.

Table 4: Initial five-factor solution for the Ethiopian dataset (N=6,183)

Question content	F1	F2	F3	F4	F5	Communality	Uniqueness
If a family can afford for one child to go to secondary school it should be the boy only	0.53	0.08	0.01	0.03	-0.07	0.33	0.67
Only boys should learn about science, technology, and math	0.61	-0.02	0.00	0.00	-0.05	0.37	0.63
Girls should be sent to school only if they are not needed to help at home	0.69	-0.04	-0.01	-0.04	-0.02	0.46	0.54
Girls should avoid raising their voice to be lady like	0.25	0.22	0.01	0.06	-0.04	0.16	0.84
Boys should be able to show their feelings without fear of being teased	0.07	-0.14	0.01	0.10	0.06	0.03	0.97
Girls and boys should share household tasks equally	0.07	0.04	-0.02	0.31	0.01	0.11	0.89
A woman's most important role is to take care of her home and cook for her family	0.17	0.45	-0.02	-0.01	0.02	0.28	0.72
A man should have the final word on decisions in his home	0.11	0.56	-0.01	0.01	-0.02	0.38	0.62
Women should have the same chance to work outside of the home as men	0.13	-0.08	-0.05	0.43	0.03	0.20	0.80
Women who participate in politics or leadership positions cannot also be good wives or mothers	0.36	0.12	0.05	-0.02	-0.05	0.19	0.81
It is important for women and adolescent girls to have their own savings	0.26	-0.18	-0.06	0.23	0.05	0.13	0.87
A boy should be able to have a girlfriend if he wants to	-0.02	-0.02	0.00	0.00	0.88	0.78	0.22
A girl should be able to have a boyfriend if she wants to	0.03	0.03	0.00	0.01	0.88	0.78	0.22
A girl's marriage can wait until she has completed secondary school	0.19	-0.16	-0.04	0.07	0.05	0.05	0.95
Girls should be proud of their bodies as they become women	-0.03	-0.01	-0.11	0.04	0.12	0.03	0.97
Families should control their daughters' behaviors more than their sons	0.10	0.05	0.57	0.00	-0.07	0.38	0.62

Our culture makes it harder for girls to achieve their goals than boys	0.32	0.17	0.04	0.12	-0.02	0.20	0.80
Adolescent girls in my community are more likely to be out of school than adolescent boys	0.17	0.17	0.08	0.07	0.03	0.10	0.90
Girls in my community are sent to school only if they are not needed to help at home	0.66	0.01	0.01	0.00	0.07	0.44	0.56
Most people in my community expect girls to be sent to school only if they are not needed at home	0.55	0.03	0.03	-0.05	0.07	0.32	0.68
Most boys and girls in my community do not share household tasks equally	-0.03	0.33	0.07	0.01	-0.03	0.13	0.87
Most people in my community expect men to have the final word about decisions in the home	-0.05	0.68	0.00	0.02	0.01	0.45	0.55
Most people in my community do not expect girls and boys to share household tasks equally	0.00	0.50	0.00	-0.09	0.02	0.25	0.75
Most men in my community are the ones who make the decisions in their home	-0.05	0.67	0.02	0.00	0.01	0.43	0.57
Most women in my community have the same chance to work outside the home as men	-0.02	0.00	0.00	0.80	-0.01	0.64	0.36
Most people in my community expect women to have the same chance to work outside the home as men	-0.03	0.02	0.02	0.75	0.01	0.56	0.44
Most adolescent girls in my community marry before the age of 18 years (legal age)	0.12	0.31	0.03	0.19	-0.04	0.20	0.80
Adults in my community expect adolescent girls to get married before the age of 18 years (legal age)	0.14	0.31	0.02	0.16	-0.04	0.19	0.81
Most families in my community control their daughters' behaviors more than their sons'	0.01	-0.03	0.92	0.00	0.00	0.82	0.18
Most people in my community expect families to control their daughter's behavior more than their sons'	-0.01	0.01	0.86	0.00	0.02	0.75	0.25
Cumulative variance explained	8.44%	16.24%	22.84%	28.42%	33.82%		

Communality and uniqueness are rounded to the second decimal place
Green highlighted factor loadings = above 0.50 (strong loading)
Yellow highlighted factor loadings = above 0.30 (weak loading)
Grey highlighted rows = community-level gender norms

Although the communalities in this factor solution were somewhat higher than in the two-factor solution, a number of variables with very low communalities remain. Furthermore, although the number of unassigned variables is lower than in the two-factor solution, six

variables remain that do not load onto any factor. There now is a higher number of variables with uniqueness values below 0.6, but 13 variables remain with values around 0.8 and higher. And while the cumulative proportion of variance explained is higher than for either two-factor solution at 33.82%, this proportion is still below the desired minimum of 50% (31).

In the Bangladeshi dataset, a similar picture emerges, with the five factors consisting mainly of *education*, *sexual and reproductive health*, *marriage and relationship*, *time-use*, and *financial and economic empowerment variables*, respectively. Ten variables did not load onto any factor. The five-factor solution for the Bangladeshi dataset is shown in Table 5. A number of variables with very low communalities remain; 12 variables had a uniqueness above 0.8 and only eight variables had a uniqueness below 0.6. The cumulative proportion of variance explained was only 29.84%.

Table 5: Initial five-factor solution for the Bangladeshi dataset (N=2,245)

Question content	F1	F2	F3	F4	F5	Communality	Uniqueness
If a family can afford for one child to go to secondary school it should be the boy only	0.35	-0.05	0.05	0.19	-0.01	0.19	0.81
Only boys should learn about science, technology, and math	0.40	-0.06	0.02	0.19	-0.03	0.24	0.76
Girls should be sent to school only if they are not needed to help at home	0.61	-0.06	0.02	0.07	0.02	0.41	0.59
Girls should avoid raising their voice to be lady like	-0.23	0.11	-0.18	0.23	0.03	0.14	0.86
Boys should be able to show their feelings without fear of being teased	-0.19	-0.03	0.06	-0.21	0.06	0.11	0.89
Girls and boys should share household tasks equally	0.10	-0.01	0.06	-0.04	0.15	0.04	0.96
A woman’s most important role is to take care of her home and cook for her family	0.04	0.00	-0.04	0.54	0.07	0.33	0.67
A man should have the final word on decisions in his home	0.13	-0.02	0.04	0.58	-0.02	0.38	0.62
Women should have the same chance to work outside of the home as men	0.08	-0.04	0.13	0.11	0.41	0.22	0.78
Women who participate in politics or leadership positions cannot also be good wives or mothers	0.03	0.01	-0.03	0.23	0.03	0.07	0.93

It is important for women and adolescent girls to have their own savings	0.15	-0.10	0.07	0.08	0.17	0.08	0.92
A boy should be able to have a girlfriend if he wants to	0.00	0.00	0.89	-0.01	0.01	0.80	0.20
A girl should be able to have a boyfriend if she wants to	-0.02	0.02	0.88	0.02	0.00	0.76	0.24
A girl's marriage can wait until she has completed secondary school	0.26	-0.05	0.20	-0.23	0.01	0.14	0.86
Girls should be proud of their bodies as they become women	0.01	-0.04	0.19	-0.16	0.01	0.08	0.92
Families should control their daughters' behaviors more than their sons	0.04	0.60	0.05	0.00	-0.06	0.36	0.64
Our culture makes it harder for girls to achieve their goals than boys	0.14	0.20	0.01	0.01	-0.06	0.07	0.93
Adolescent girls in my community are more likely to be out of school than adolescent boys	0.39	0.13	0.14	-0.18	-0.07	0.18	0.82
Girls in my community are sent to school only if they are not needed to help at home	0.69	-0.01	-0.02	0.03	0.03	0.49	0.51
Most people in my community expect girls to be sent to school only if they are not needed at home	0.61	0.01	-0.07	0.05	0.00	0.40	0.60
Most boys and girls in my community do not share household tasks equally	-0.01	0.28	0.04	0.04	-0.02	0.08	0.92
Most people in my community expect men to have the final word about decisions in the home	0.02	0.05	0.02	0.57	0.03	0.36	0.64
Most people in my community do not expect girls and boys to share household tasks equally	0.01	0.15	0.03	0.06	-0.12	0.04	0.96
Most men in my community are the ones who make the decisions in their home	-0.03	0.12	-0.02	0.54	-0.04	0.33	0.67
Most women in my community have the same chance to work outside the home as men	0.01	0.03	-0.02	-0.02	0.71	0.50	0.50
Most people in my community expect women to have the same chance to work outside the home as men	-0.01	-0.01	0.00	0.00	0.76	0.57	0.43
Most adolescent girls in my community marry before the age of 18 years (legal age)	0.33	0.30	-0.09	-0.11	-0.01	0.19	0.81
Adults in my community expect adolescent girls to get married before the age of 18 years (legal age)	0.34	0.24	-0.11	-0.09	0.03	0.17	0.83
Most families in my community control their daughters' behaviors more than their sons'	-0.01	0.78	0.03	0.01	0.02	0.62	0.38

Most people in my community expect families to control their daughter's behavior more than their sons'	-0.03	0.75	-0.03	0.04	0.02	0.58	0.42
Cumulative variance explained	7.26%	13.64%	19.57%	25.34%	29.84%		

Communality and uniqueness are rounded to the second decimal place
Green highlighted factor loadings = above 0.50 (strong loading)
Yellow highlighted factor loadings = above 0.30 (weak loading)
Grey highlighted rows = community-level gender norms

These results support the conclusion that dividing the variables by domain is more appropriate than dividing them into individual-level and community-level variables. However, the five-factor solutions remain far from perfect in both datasets, exhibiting low communalities, non-loading variables and low proportions of explained variance.

These issues could indicate that a number of factors other than two or five may provide a better factor solution. However, they may also reflect issues with the original data, and the need to reconsider the wording or domain assignment of some questions. Therefore, we next investigated whether removing problematic items improves the five-factor solutions in the Ethiopian and Bangladeshi datasets.

3.2.3 Refined Five-Factor Solution

To refine the initial five-factor solution for Ethiopia, we removed six variables that failed to load onto any factor (cr_fin_impwsav, cr_edu_raisingvoice, cr_mar_waitedu, cr_edu_eegirlsout, cr_edu_boysfeelings, cr_srh_proudbod). This slightly reduced the KMO from 0.77 to 0.75. Thereafter, we removed another three variables for the same reason (cr_tu_statements1, cr_mar_eemarryage, cr_mar_nemarryage), which resulted in all variables loading onto at least one factor. This did not change the KMO. Finally, we excluded four more variables with low communalities and factor loadings (cr_fin_notgood, cr_fin_girlchance, cr_edu_culture, cr_tu_statements4), resulting in a KMO of 0.71. The final set of 17 variables is shown in Table 6.

The refined five-factor solution shows that each factor corresponds to exactly one of the five domains. The first factor contains five *education* variables with four of them loading strongly onto the factor. The second factor is strongly loaded onto by three *sexual and reproductive*

health variables while the third factor is loaded onto by five *time-use* variables, with most of them loading strongly. The fourth and fifth factors only include two variables each. The two *marriage and relationship* variables loaded strongly onto the fourth and the two *financial and economic empowerment* variables loaded strongly onto the fifth factor.

Table 6: Refined five-factor solution for the Ethiopian dataset (N=6,183)

Question content	F1	F2	F3	F4	F5	Communality	Uniqueness
If a family can afford for one child to go to secondary school it should be the boy only	0.50	0.01	0.08	-0.07	0.04	0.29	0.71
Only boys should learn about science, technology, and math	0.58	-0.01	0.01	0.06	0.01	0.35	0.65
Girls should be sent to school only if they are not needed to help at home	0.71	-0.02	-0.03	-0.03	-0.02	0.49	0.51
A woman's most important role is to take care of her home and cook for her family	0.17	-0.01	0.45	0.02	-0.03	0.28	0.72
A man should have the final word on decisions in his home	0.09	0.00	0.62	-0.02	-0.02	0.42	0.58
A boy should be able to have a girlfriend if he wants to	-0.02	0.00	-0.02	0.89	0.00	0.80	0.20
A girl should be able to have a boyfriend if she wants to	0.02	0.00	0.03	0.88	0.00	0.77	0.23
Families should control their daughters' behaviors more than their sons	0.07	0.57	0.06	-0.07	-0.04	0.38	0.62
Girls in my community are sent to school only if they are not needed to help at home	0.70	0.01	-0.01	0.06	0.03	0.49	0.51
Most people in my community expect girls to be sent to school only if they are not needed at home	0.57	0.03	0.02	0.06	-0.03	0.34	0.66
Most people in my community expect men to have the final word about decisions in the home	-0.05	0.00	0.73	0.01	0.03	0.51	0.49
Most people in my community do not expect girls and boys to share household tasks equally	0.04	0.01	0.47	0.02	-0.05	0.23	0.77
Most men in my community are the ones who make the decisions in their home	-0.05	0.02	0.69	0.00	0.01	0.47	0.53
Most women in my community have the same chance to work outside the home as men	0.01	-0.01	0.00	-0.01	0.81	0.65	0.35
Most people in my community expect women to have the same chance to work outside the home as men	0.00	0.01	0.00	0.01	0.81	0.66	0.34

Most families in my community control their daughters' behaviors more than their sons'	-0.01	0.91	-0.03	0.00	0.01	0.82	0.18
Most people in my community expect families to control their daughter's behavior more than their sons'	-0.01	0.86	0.01	0.01	0.00	0.75	0.25
Cumulative variance explained	11.74%	23.05%	34.03%	43.38%	51.12%		

Communality and uniqueness are rounded to the second decimal place
Green highlighted factor loadings = above 0.50 (strong loading)
Yellow highlighted factor loadings = above 0.30 (weak loading)
Grey highlighted rows = community-level gender norms

Although some communalities remain relatively low in the refined five-factor solution, the lowest value is now 0.23. While six variables have uniqueness values higher than 0.6, none exceed 0.8. The cumulative proportion of variance explained with over 51% is noticeably higher than in the initial five-factor solution.

The results in the Bangladeshi dataset, displayed in Table 7, show a similar picture. After removing ten variables that failed to load onto any of the five factors(cr_edu_raisingvoice, cr_edu_boysfeelings, cr_tu_statements1, cr_fin_notgood, cr_fin_impwsav, cr_mar_waitedu, cr_srh_proudbod, cr_edu_culture, cr_tu_statements4, cr_tu_statements6), we further excluded five variables with low communalities and factor loadings(cr_edu_boysch, cr_fin_girlchance, cr_edu_eegirlsout, cr_mar_eemarryage, cr_mar_nemarryage). This process resulted in a final set of 15 variables, all of which loaded onto one of the factors. The KMO decreased from 0.74 and 0.72 to 0.69, which remains an acceptable, but not ideal value to conduct EFAs.

The refined five-factor solution contains three *sexual and reproductive health* variables that loaded strongly onto the first factor. All four *education* variables loaded onto the second factor (three of them strongly), while two *marriage and relationship* variables loaded strongly onto the third factor. Four *time-use* variables loaded onto the fourth factor, with three of them loading strongly, while *two financial and economic empowerment* variables loaded strongly onto the fifth factor.

Table 7: Refined five-factor solution for the Bangladeshi dataset (2,245)

Question content	F1	F2	F3	F4	F5	Communality	Uniqueness

Only boys should learn about science, technology, and math	-0.01	0.40	0.01	0.14	-0.04	0.22	0.78
Girls should be sent to school only if they are not needed to help at home	-0.03	0.66	0.03	0.02	0.01	0.45	0.55
A woman's most important role is to take care of her home and cook for her family	0.00	0.05	-0.05	0.50	0.05	0.29	0.71
A man should have the final word on decisions in his home	-0.02	0.10	0.02	0.59	-0.04	0.38	0.62
A boy should be able to have a girlfriend if he wants to	-0.01	0.01	0.90	-0.02	0.01	0.81	0.19
A girl should be able to have a boyfriend if she wants to	0.01	-0.01	0.90	0.02	-0.01	0.81	0.19
Families should control their daughters' behaviors more than their sons	0.61	0.02	0.04	0.02	-0.08	0.38	0.62
Girls in my community are sent to school only if they are not needed to help at home	0.02	0.78	0.00	-0.03	0.01	0.58	0.42
Most people in my community expect girls to be sent to school only if they are not needed at home	0.01	0.62	-0.04	0.03	0.00	0.41	0.59
Most people in my community expect men to have the final word about decisions in the home	-0.01	-0.02	0.02	0.66	0.04	0.43	0.57
Most men in my community are the ones who make the decisions in their home	0.06	-0.07	-0.02	0.61	-0.02	0.36	0.64
Most women in my community have the same chance to work outside the home as men	0.01	0.00	-0.01	-0.01	0.75	0.56	0.44
Most people in my community expect women to have the same chance to work outside the home as men	-0.01	0.00	0.01	0.01	0.76	0.57	0.43
Most families in my community control their daughters' behaviors more than their sons'	0.84	0.00	0.02	-0.01	0.01	0.71	0.29
Most people in my community expect families to control their daughter's behavior more than their sons'	0.79	0.00	-0.04	0.01	0.02	0.63	0.37
Cumulative variance explained	11.41 %	22.32 %	33.22 %	42.93 %	50.57 %		

Communality and uniqueness are rounded to the second decimal place
Green highlighted factor loadings = above 0.50 (strong loading)
Yellow highlighted factor loadings = above 0.30 (weak loading)
Grey highlighted rows = community-level gender norms

The communalities and uniqueness values are comparable to the Ethiopian factor solution. No very low communalities and no uniqueness values higher than 0.8 remain. Only five uniqueness values are slightly above 0.6. As in the Ethiopian dataset, the cumulative variance accounts for an acceptable proportion of 50.57%.

Table 8: Comparison of the refined five-factor structure with variables loading significantly onto a factor in the Ethiopian and the Bangladeshi datasets

	Ethiopia					Bangladesh				
Question content	F1	F2	F3	F4	F5	F1	F2	F3	F4	F5
Education domain										
If a family can afford for one child to go to secondary school it should be the boy only										
Only boys should learn about science, technology, and math										
Girls should be sent to school only if they are not needed to help at home										
Girls in my community are sent to school only if they are not needed to help at home										
Most people in my community expect girls to be sent to school only if they are not needed at home										
Time use domain										
A woman's most important role is to take care of her home and cook for her family										
A man should have the final word on decisions in his home										
Most people in my community expect men to have the final word about decisions in the home										
Most people in my community do not expect girls and boys to share household tasks equally										
Most men in my community are the ones who make the decisions in their home										
Financial and economic empowerment domain										
Most women in my community have the same chance to work outside the home as men										
Most people in my community expect women to have the same chance to work outside the home as men										
Marriage and relationship domain										
A boy should be able to have a girlfriend if he wants to										
A girl should be able to have a boyfriend if she wants to										
Sexual and reproductive health domain										
Families should control their daughters' behaviors more than their sons										
Most families in my community control their										

daughters' behaviors more than their sons'										
Most people in my community expect families to control their daughter's behavior more than their sons'										

Grey background = perceived community-level gender norms
White background = gender attitudes
Green highlighted factor loadings = above 0.50 (strong loading)
Yellow highlighted factor loadings = above 0.30 (weak loading)

Comparing the refined five-factor solutions in Table 8, each factor corresponds exactly to one of the five domains. With the exception of the two additional variables in the Ethiopian dataset and one variable that loads weaker in the Bangladeshi dataset, the same variables load with the same strength onto the factors. Table 8 also shows that individual-level gender attitudes and community-level gender norms are mixed within the domains (at least where a sufficient number of variables is available in the data). This further supports the notion that the individual-community distinction is not suitable for the available GAGE gender norms items in Ethiopia and Bangladesh.

4 Discussion

4.1 Principal Findings

To assess whether the items and domains of the GAGE gender norms scale contribute to explaining gender norms and attitudes in Ethiopia and Bangladesh, we explored the implied two- and five-factor structures using EFA. The two-factor structure, distinguishing individual-level gender attitudes and community-level gender norms, showed an unacceptable model fit in both datasets. In contrast, the initial five-factor solution showed indications of a five-domains structure (*education; sexual and reproductive health; relationships and marriage; time use; and financial and economic empowerment*). After having refined the five-factor solution, we found that the variables captured their respective domains. However, this was only the case for a reduced set of gender norms items in both datasets. Therefore, we propose an adaptation of the GAGE gender norms scale for Ethiopia and Bangladesh.

Concerning the inadequate two-factor solution, we hypothesise that the distinction between gender attitudes and gender norms in this dataset is not substantial enough to yield two factors in the EFA. This may stem from the community-level gender norms being reported by adolescents themselves, rather than other community members. Consequently, their responses likely reflect their own perspective on community norms rather than the actual norms within the community.

Evidence for a two-factor solution of gender norms items has been observed in the Nepali context, with the G-Norm scale differentiating between descriptive and injunctive norms (32). The G-NORM scale has been incorporated into the GAGE gender norms scale, which also contains descriptive and injunctive norms (23). However, Baird et al.'s analysis does not account for the descriptive-injunctive distinction, instead creating separate scores for individual-level gender attitudes and perceived community-level gender norms.

A recent study among adolescents in Bangladesh also successfully validated a multi-domain structure for a 'gender norms attitude' scale (M-GNAS) (3). This four-domain structure includes 13 items and covers the areas of gender attitudes identified as dominant among Bangladeshi youth: gender-appropriate behaviour, family financial decisions, family responsibility, and career choice (3). Islam et al., (2024) (3) highlighted that the lack of women's empowerment in decision-making and deeply entrenched gender norms within the family and socioeconomic context are specific to the Bangladeshi context. This makes the *time-use* variables in the GAGE gender norms scale particularly important.

The exclusion of certain items in the refined five-factor solutions may indicate conceptual issues in the GAGE gender norms scale. One reason could be that these items do not align closely enough with the content represented in the factor structures. The item 'cr_edu_boysfeelings', for example, asks, whether "boys should be able to show their feelings without the fear of being teased". This statement does not only apply to a school context, nor is it similar to the other school-related questions. Yet, the item was assigned to the *education* domain. Furthermore, this item contains two questions in one – whether boys

should be able to show their feelings and whether they should be able to do so without fear of being teased. This makes it challenging to interpret the responses. Another conceptual issue is the discrepancy between the content of the items and the domains. In particular, the *sexual and reproductive health* domain includes one item about girls' bodies in their transition to womanhood, but the remaining items ask about control, which is not specifically related to sexual and reproductive health. Although sexual and reproductive health questions should be asked with caution when surveying young adolescents, this domain does not cover the content it suggests.

Another limitation of the GAGE gender norms scale could be its focus on a binary understanding of sex, along with gendered questions that are only applicable to one of the two sexes. This conceptual aspect likely applies to most existing gender norms scales. Nevertheless, gender norms could be assessed in a way that makes them applicable to both or all genders. Moreover, gender norms and attitudes in the GAGE gender norms scale are sometimes measured indirectly through other constructs, such as gender roles, gender stereotypes, and traits (3). For example, items in the *time-use* domain, such as "girls and boys should share household tasks equally", may relate more to the construct of gender roles than gender norms. This reflects the inconsistency in the definition of gender norms, the conflation of terms, and the challenges in the conceptualisation of gender-related aspects in the scientific literature.

Comparing the refined five-factor solutions for Ethiopia and Bangladesh, we found that the 15 gender norms items in Bangladesh are the same 15 gender norms items in Ethiopia, with two additional items specific to the Ethiopian dataset. This demonstrates that a subset of gender norms items was retained consistently across two different geographical contexts. We hypothesise that these variables may be more robust across cultures. Although further research is needed, these variables could serve as a basis for improving the GAGE gender norms scale and for facilitating cross-cultural comparisons. In contrast, the remaining 17 and 15 variables out of the original 30 items indicate that up to half of the items may not be well-

suited for the GAGE gender norms scale in Ethiopia and Bangladesh. These items may need to be refined to enhance their alignment with the five-domain structure. This underscores the importance of validating the scale for each cultural context (3, 33). Our results may be generalisable to countries with a similar cultural context to Ethiopia and Bangladesh.

4.2 Strengths and limitations

In our study, we followed methodological recommendations in performing our EFA by using PFA as the conceptually desirable method compared to PCA (34, 27), by using oblique rather than orthogonal rotation due to the high correlations among the gender norms items (30), by using relatively large samples (which are particularly important in EFAs) (30) from two LMI countries, and by conducting several factor models (35). The latter increases the likelihood that the results can be generalised if the same factor structures are retrieved (30). This may be the case for 15 gender norms items in our analysis.

The limitations of our study are twofold. First, there are limitations in the GAGE datasets which we rely on in our secondary data analysis. Despite being administered by skilled and trained interviewers, the GAGE data may still be influenced by social desirability and other biases, such as measurement invariance. Additionally, the GAGE gender norms items reflect perceived rather than objective measures. Also, some items in the scale may need to be rephrased to produce more valid outcomes.

Furthermore, the provided response options – ‘agree’, ‘partially agree’ and ‘disagree’ - are unbalanced due to the absence of a weak negative response option, such as ‘partially disagree’. Recent gender norms scales employ five-point Likert scales, which provide an additional neutral response option (3, 32). Additionally, two factors in the refined five-factor solution (namely *marriage and relationship* and *financial and economic empowerment* norms) each consist of only two variables in both datasets. This is usually considered an insufficient amount for factor analysis. The GAGE data may benefit from additional variables in both domains.

Second, our analysis has its own methodological limitations. We did not try other rotation and extraction methods other than oblimin principal axis factoring in our EFA. Lastly, we conducted EFA and not Confirmatory Factor Analysis (CFA). Besides further refining the GAGE gender norms items, conducting the CFA is a necessary next step to validate the scale.

4.3 Conclusion

Gender norms research faces a number of challenges in defining, measuring, and conceptualising gender norms. This applies particularly to gender norms scales for adolescents. Our results indicate that separating the GAGE gender norms items into individual-level gender attitudes and community-level gender norms may not reflect the data structure for the Ethiopia and Bangladesh datasets. Instead, differentiating into different domains seems more promising. In fact, only our refined five-factor solutions yielded promising results. However, there are still issues in the GAGE gender norms items, such as the wording or the domain assignment of some questions. With our EFAs, we contribute to improving the validity of a gender norms scale for adolescents in two LMI countries. A refined gender norms scale could be valuable for developing more effective interventions in Ethiopia and Bangladesh to reduce unequal gender norms.

5 References

1. Khan A, Khan S, Khan MA, Zaman K, Khan HUR, Rosman ASB et al. Economic costs of gender inequality in health and the labor market: India's untapped potential. Frontiers in Public Health 2023; 11:1067940. Available from: URL: https://pmc.ncbi.nlm.nih.gov/articles/PMC9922756/.

2. Weber AM, Cislaghi B, Meausoone V, Abdalla S, Mejía-Guevara I, Loftus P et al. Gender norms and health: insights from global survey data. The Lancet 2019; 393(10189):2455–68.

3. Islam A, Anwar Siraji M, Haque M, Salim Chowdhury M. Development of a multidomain gender norm attitude scale for youth in Bangladesh. Preventive Medicine Reports 2024; 45:102848. Available from: URL: https://www.sciencedirect.com/science/article/pii/S2211335524002638.

4. Hill AL, Miller E, Switzer GE, Abebe KZ, Chang JC, Pulerwitz J et al. Gender Equitable Attitudes Among Adolescents: A Validation Study and Associations with Sexual Health Behaviors. Adolescent research review 2022; 7(4):523–36. Available from: URL: https://www.ncbi.nlm.nih.gov/pmc/articles/PMC11185410/.

5. Weziak-Bialowolska D. Differences in Gender Norms Between Countries: Are They Valid? The Issue of Measurement Invariance. Eur J Popul 2015; 31(1):51–76. Available from: URL: https://www.ncbi.nlm.nih.gov/pmc/articles/PMC4315909/.

6. Weber AM, Gupta R, Abdalla S, Cislaghi B, Meausoone V, Darmstadt GL. Gender-related data missingness, imbalance and bias in global health surveys. BMJ Glob Health 2021; 6(11):e007405. Available from: URL: https://www.researchgate.net/publication/356574489_Gender-related_data_missingness_imbalance_and_bias_in_global_health_surveys.

7. Lundgren R, Beckman M, Chaurasiya SP, Subhedi B, Kerner B. Whose turn to do the dishes? Transforming gender attitudes and behaviours among very young adolescents in Nepal. Gender & Development 2013; 21(1):127–45.

8. Levtov RG, Vlahovicova K, Barker G, Stiefvater E, Daimon L, Mulokozi AD. Momentum toward equality: Results from the international men and gender equality survey (IMAGES) in Tanzania.; 2018. Available from: URL: https://www.equimundo.org/wp-content/uploads/2018/10/Momentum-Toward-Equality-IMAGES-Tanzania-Report-EN-POSTPRINT-2.25.19.pdf.

9. Sedlander E, Bingenheimer JB, Long MW, Swain M, Rimal RN. The G-NORM Scale: Development and Validation of a Theory-Based Gender Norms Scale. Sex Roles 2022; 87(5-6):350–63. Available from: URL: https://www.ncbi.nlm.nih.gov/pmc/articles/PMC9508194/.

10. Moreau C, Li M, Meyer S de, Vu Manh L, Guiella G, Acharya R et al. Measuring gender norms about relationships in early adolescence: Results from the global early adolescent study. SSM - Population Health 2019; 7:14. Available from: URL: https://www.ncbi.nlm.nih.gov/pmc/articles/PMC6293033/.

11. Hossain MM, Nesa F, Das J, Aggad R, Tasnim S, Bairwa M et al. Global burden of mental health problems among children and adolescents during COVID-19 pandemic: An umbrella review. Psychiatry Res 2022; 317:114814. Available from: URL: https://www.ncbi.nlm.nih.gov/pmc/articles/PMC9420079/.

12. Thomson KC, Romaniuk H, Greenwood CJ, Letcher P, Spry E, Macdonald JA et al. Adolescent antecedents of maternal and paternal perinatal depression: a 36-year prospective cohort. Psychol Med 2021; 51(12):2126–33.

13. World Health Organization. Mental health of adolescents; 2021. Available from: URL: https://www.who.int/news-room/fact-sheets/detail/adolescent-mental-health.

14. Blum RW, Mmari K, Moreau C. It Begins at 10: How Gender Expectations Shape Early Adolescence Around the World. J Adolesc Health 2017; 61(4 Suppl):S3-S4. Available from: URL: https://www.sciencedirect.com/science/article/pii/S1054139X17303555.

15. Saeed Ali T, Karmaliani R, Mcfarlane J, Khuwaja HMA, Somani Y, Chirwa ED et al. Attitude towards gender roles and violence against women and girls (VAWG): baseline findings from an RCT of 1752 youths in Pakistan. Global Health Action 2017; 10(1):1342454. Available from: URL: https://www.ncbi.nlm.nih.gov/pmc/articles/PMC5964875/.

16. Vu L, Pulerwitz J, Burnett-Zieman B, Banura C, Okal J, Yam E. Inequitable Gender Norms From Early Adolescence to Young Adulthood in Uganda: Tool Validation and Differences Across Age Groups. J Adolesc Health 2017; 60(2S2):S15-S21. Available from: URL: https://www.sciencedirect.com/science/article/pii/S1054139X16303767.

17. Galambos NL, Petersen AC, Richards M, Gitelson IB. The Attitudes Toward Women Scale for Adolescents (AWSA): A study of reliability and validity. Sex Roles 1985; 13(5-6):343–56.

18. Tolman DL, Porche MV. The Adolescent Femininity Ideology Scale: Development and Validation of a New Measure for Girls. Psychology of Women Quarterly 2000; 24(4):365–76.

19. Pulerwitz J, Barker G. Measuring Attitudes toward Gender Norms among Young Men in Brazil. Men and Masculinities 2008; 10(3):322–38.

20. GEAS quantitative measures; 2018. Available from: URL: https://www.geastudy.org/quant-download/.

21. Levant RF, McDermott RC, Hewitt AA, Alto KM, Harris KT. Confirmatory factor analytic investigation of variance composition, gender invariance, and validity of the Male Role Norms Inventory-Adolescent-revised (MRNI-A-r). J Couns Psychol 2016; 63(5):543–56.

22. Beckwith S, Li M, Barker KM, Gayles J, Kågesten AE, Lundgren R et al. The Impacts of Two Gender-Transformative Interventions on Early Adolescent Gender Norms Perceptions: A Difference-in-Difference Analysis. J Adolesc Health 2023; 73(1S):S55-S64.

23. Baird S, Bhutta ZA, Hamad BA, Hicks JH, Jones N, Muz J. Do restrictive gender attitudes and norms influence physical and mental health during very young Adolescence? Evidence from Bangladesh and Ethiopia. SSM - Population Health 2019; 9:100480. Available from: URL: https://www.sciencedirect.com/science/article/pii/S2352827318302520.

24. Kallgren CA, Reno RR, Cialdini RB. A Focus Theory of Normative Conduct: When Norms Do and Do not Affect Behavior. Pers Soc Psychol Bull 2000; 26(8):1002–12.

25. Hamory J, Jones N, Woldehanna T, Baird S. GAGE Ethiopia Round 2 Quantitative Research Design and Sample. London; 2023. Quantitative Research Manual Series.

26. Seager J, Jones N, Baird S, Haque MA. GAGE Bangladesh-Chittagong and Sylhet 2018 Cross Section Basic Information Document. London; 2022. Quantitative Research Manual Series.

27. Osborne JW. Best Practices in Exploratory Factor Analysis; 2014 7. Available from: URL: https://www.researchgate.net/publication/209835856_Best_Practices_in_Exploratory_Factor_Analysis_Four_Recommendations_for_Getting_the_Most_From_Your_Analysis.

28. Child D. The essentials of factor analysis. 3. ed. London: Continuum; 2006.

29. Samuels P. Advice on Exploratory Factor Analysis. Birmingham City University; 2016.

30. Field A. Discovering statistics using SPSS: (and sex and drugs and rock 'n' roll). 3. ed. Los Angeles: Sage Publ; 2009.

31. Streiner DL. Figuring out factors: the use and misuse of factor analysis. Canadian journal of psychiatry. Revue canadienne de psychiatrie 1994; 39(3):135–40. Available from: URL: https://pubmed.ncbi.nlm.nih.gov/8033017/.

32. Sedlander E, Dahal M, Bingenheimer JB, Puri MC, Rimal RN, Granovsky R et al. Adapting and Validating the G-NORM (Gender Norms Scale) in Nepal: An Examination of How Gender Norms Are Associated with Agency and Reproductive Health Outcomes. Studies in family planning 2023; 54(1):181–200.

33. Seddig D, Lomazzi V. Using cultural and structural indicators to explain measurement noninvariance in gender role attitudes with multilevel structural equation modeling. Social Science Research 2019; 84:102328. Available from: URL: https://www.sciencedirect.com/science/article/pii/S0049089X18309724.

34. Gorsuch RL. Common Factor Analysis versus Component Analysis: Some Well and Little Known Facts. Multivariate Behavioral Research 1990; 25(1):33–9.

35. Watts AL, Greene AL, Ringwald W, Forbes MK, Brandes CM, Levin-Aspenson HF et al. Factor analysis in personality disorders research: Modern issues and illustrations of practical recommendations. Personality disorders 2023; 14(1):105–17. Available from: URL: https://www.ncbi.nlm.nih.gov/pmc/articles/PMC10044462/.
